# Monolayer-to-thin-film transition in supramolecular assemblies: the role of topological protection†
†Electronic supplementary information (ESI) available: AFM images, STM images, TEM diffraction and acTEM images, multislice TEM image simulation details, and theoretical derivations of acTEM image reconstruction. See DOI: 10.1039/c7nr03588h. All data presented in this paper are available at http://wrap.warwick.ac.uk/90210.


**DOI:** 10.1039/c7nr03588h

**Published:** 2017-08-01

**Authors:** Zachary P. L. Laker, Alexander J. Marsden, Oreste De Luca, Ada Della Pia, Luís M. A. Perdigão, Giovanni Costantini, Neil R. Wilson

**Affiliations:** a Department of Physics , University of Warwick , Coventry , CV4 7AL , UK . Email: Neil.Wilson@warwick.ac.uk; b Department of Chemistry , University of Warwick , Coventry , CV4 7AL , UK . Email: G.Costantini@warwick.ac.uk; c National Graphene Institute , School of Materials , University of Manchester , Manchester , M13 9PL , UK; d Dipartimento di Fisica , Università della Calabria , 87036 Arcavacata di Rende (CS) , Italy

## Abstract

The ability to control the transition from a two-dimensional (2D) monolayer to the three-dimensional (3D) molecular structure in the growth of organic layers on surfaces is essential for the production of functional thin films and devices. This has, however, proved to be extremely challenging, starting from the currently limited ability to attain a molecular scale characterization of this transition. Here, through innovative application of low-dose electron diffraction and aberration-corrected transmission electron microscopy (acTEM), combined with scanning tunneling microscopy (STM), we reveal the structural changes occurring as film thickness is increased from monolayer to tens of nanometers for supramolecular assembly of two prototypical benzenecarboxylic acids – terephthalic acid (TPA) and trimesic acid (TMA) – on graphene. The intermolecular hydrogen bonding in these molecules is similar and both form well-ordered monolayers on graphene, but their structural transitions with film thickness are very different. While the structure of TPA thin films varies continuously towards the 3D lattice, TMA retains its planar monolayer structure up to a critical thickness, after which a transition to a polycrystalline film occurs. These distinctive structural evolutions can be rationalized in terms of the topological differences in the 3D crystallography of the two molecules. The templated 2D structure of TPA can smoothly map to its 3D structure through continuous molecular tilting within the unit cell, whilst the 3D structure of TMA is topologically distinct from its 2D form, so that only an abrupt transition is possible. The concept of topological protection of the 2D structure gives a new tool for the molecular design of nanostructured films.

## Abstract 2

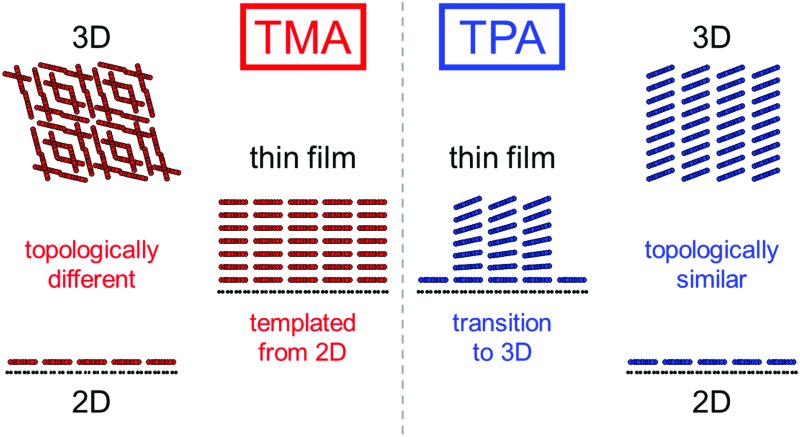
An innovative combination of TEM and STM sheds new insight into the growth of organic layers and reveals the importance of topology in controlling the transition from two- to three-dimensional structure.

## Introduction

Supramolecular assembly is a well-established route for the controlled synthesis of nanomaterials, utilizing non-covalent forces to direct the assembly of complex nanostructures from functional molecular precursors that can be precisely tuned through chemical design. Assembly on surfaces can result in well-ordered two-dimensional (2D) molecular crystals, with interactions with the surface stabilizing the molecular overlayer and influencing the nanoscale organization and crystallography.^1^–^3^ These structures can be further used to direct the assembly of ‘host’ molecules, acting as templates or traps for the formation of ordered arrays of molecules in subsequent layers.^4^–^7^ Such templated growth shows promise for creating nanostructured films for applications such as organic electronics and optoelectronics,^8^ or to control surface reactivity.^2^,^9^,^10^ The structure of the 2D molecular crystal, influenced by its interaction with the surface, usually differs from the preferred 3D molecular crystallography although, with increasing thickness, a molecular film will eventually adopt the 3D crystalline structure. Understanding how this transition occurs, and at what thickness, is essential since most applications of functional organic layers (*e.g.* in organic electronics, organic photovoltaics, sensors, *etc*.) rely on films with thicknesses that fall precisely into this transition regime. On the other hand, studying the 2D–3D evolution is particularly challenging because high-resolution analytical techniques that are traditionally used are optimized either for near monolayers (scanning tunneling microscopy, STM) or for thicker films (X-ray diffraction).

Supramolecular self-assembly at surfaces has been extensively investigated on single crystal metal substrates and highly oriented pyrolytic graphite (HOPG),^3^,^11^ although more recently the study of molecular assembly on crystalline 2D materials, such as graphene^12^–^14^ and hexagonal boron nitride (hBN),^15^ has become increasingly important. For example, non-covalent molecular functionalization has been widely explored as a means to controllably alter the electronic properties of graphene,^16^,^17^ either for electronic doping^18^,^19^ or in search of a usable electronic band gap.^20^,^21^ Alternatively, graphene has been proposed as an electrode material in organic electronics,^22^ with the ability to control molecular assembly to increase the crystallinity and define the orientation of the organic thin film, hence improving its electrical properties.^8^,^23^–^25^

STM has been the method of choice for resolving the 2D structure of the molecular overlayer as it allows direct, non-destructive imaging with sub-molecular resolution.^2^ However, it is limited to monolayer (or close-to-monolayer) films and is unable to resolve the crystallographic order in multilayer structures. Early studies used transmission electron microscope (TEM) based electron diffraction to probe the structure of self-assembled monolayers and free standing ultrathin molecular films, forming the basis of our understanding of structural transitions from monolayer to multilayer crystalline films.^26^–^28^ These studies were technically challenging: supramolecular assemblies are rapidly damaged by the electron beam, are extremely thin, and are typically carbon-based making it difficult to acquire TEM data with acceptable signal to noise levels. In addition, studying assembly on surfaces required fabrication of electron transparent single crystal TEM supports^26^ which was both time consuming and complicated. On the other hand, analysis in the TEM through combined diffraction and imaging experiments has in principle the potential to resolve organic structures with sub-molecular resolution.^29^,^30^ Recent technical advances in TEM, such as aberration correction for sub-angstrom resolution imaging and single electron detection cameras for low noise acquisition, are opening up new possibilities for studying molecular systems at even higher resolution.^31^ For TEM, graphene is a particularly exciting and relevant substrate as it is almost perfectly electron transparent, conductive, crystalline, strong, and stable.^32^ When grown on metal substrates, it is often atomically smooth and hence also well-suited for STM imaging, enabling direct comparison between the two techniques.

Here we study supramolecular assembly on graphene of benzene-1,4-dicarboxylic acid (terephthalic acid, TPA) and benzene-1,3,5-tricarboxylic acid (trimesic acid, TMA), two molecules with planar phenyl cores that can form intermolecular hydrogen bonds through their carboxylic moieties; both have been intensively studied as prototypical systems for 2D supramolecular assembly on graphitic substrates.^13^,^33^–^38^ We find that both molecules self-assemble on graphene to form well-ordered crystals from a 2D monolayer to thin films of several nanometer thickness. Combining STM, electron diffraction and acTEM imaging, we identify a structural transition that occurs as molecular deposition proceeds, and determine the critical thickness beyond which the film structure is no longer defined by the molecular 2D crystal at the substrate surface. We demonstrate that, despite the chemical similarity between the two molecules, their monolayer-to-thin-film transitions are dramatically different. These results have important implications for how supramolecular self-assembly can be used to design molecular structures grown from surfaces (from thin films to macroscopic crystals), and demonstrate how recent advances in TEM make it a powerful tool for studying surface-driven supramolecular self-assembly.

## Results and discussion

### Monolayer structure of TMA and TPA

**Fig. 1:**
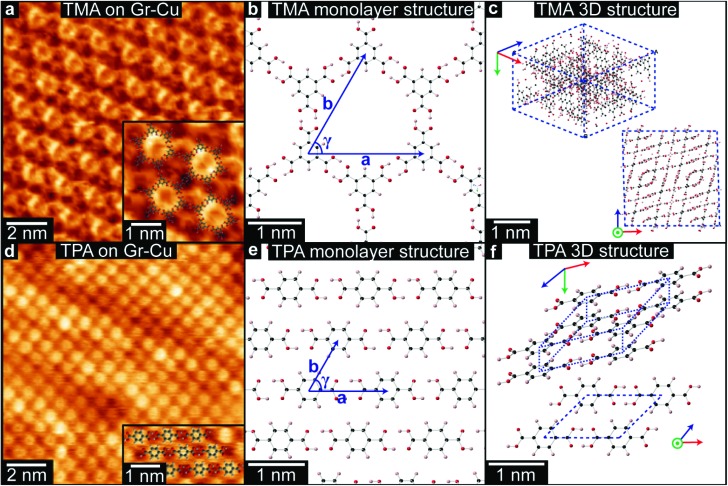
STM images of TMA (a) and TPA (d) on Gr–Cu. (Tunneling currents and voltages: (a) *I* = 50 pA, *V* = –1.3 V; (d) *I* = 80 pA, *V* = –1.5 V.) The insets show enlarged regions with superposed molecular models. Schematics of the 2D chicken-wire structure for TMA (b) and brickwork structure for TPA (e), and of the 3D structures for TMA (c) and TPA (f).

TMA and TPA were deposited onto chemical vapor deposition (CVD) grown graphene-on-copper foils (Gr–Cu, see Methods for more details) by organic molecular beam deposition (OMBD) and imaged in ambient conditions by STM at the liquid–solid interface under a drop of heptanoic acid. STM images of TMA on Gr–Cu, as in Fig. 1(a), show a hexagonal lattice, consistent with a monolayer of ‘chicken-wire’ TMA structure (shown in (b)), with lattice parameters *a* = *b* = 1.65 ± 0.06 nm and *γ* = 60 ± 1°. These are consistent with the values previously reported for TMA deposited on HOPG^34^,^35^ and on graphene.^13^,^39^ While other types of TMA assemblies have been reported on various graphite and graphene substrates,^34^,^35^,^39^,^40^ we only observed the chicken-wire packing on Gr–Cu, as also described by MacLeod *et al.*^13^ We note that the absence of other assemblies, such as the flower structure, might be due to the deposition conditions used here.

By contrast TPA packs more densely; Fig. 1(d) shows the characteristic brickwork arrangement of TPA molecules, with lattice parameters *a* = 0.95 ± 0.02 nm, *b* = 0.75 ± 0.06 nm, and *γ* = 53 ± 3°, consistent with previous reports for TPA deposited onto graphene on Pt(111).^37^ For both TPA and TMA, the supramolecular assembly on Gr–Cu is thus similar to that previously found for other graphitic samples.

The difference in the 2D supramolecular packing between TMA and TPA is driven by the difference in their chemical structure. The 3-fold symmetric carboxylic acid moieties of TMA lead to hexagonal assembly, whilst dimeric hydrogen-bonding between the two linearly-aligned carboxylic acid groups in TPA creates strongly bonded molecular rows with a weak interaction between them (Fig. 1(b) and (e)). The changes in packing are even more profound in their 3D bulk crystalline structures,^41^,^42^ as shown in Fig. 1(c) and (f). While TMA forms crystals of interweaving planes of TMA molecules that are hydrogen bonded in small units of the chicken-wire structure, for TPA the bulk structure is formed of tilted hydrogen bonded lamellar rows, that resemble quite closely the monolayer structure. Although the molecular packing is denser in the 3D structure, its projected view (Fig. 1(f)) is very similar to the 2D structure shown in Fig. 1(e).

### Resolving the structure of TMA thin-films

We use TEM analysis of TMA deposited on freestanding graphene to reveal the structural changes that occur as film thickness increases. Monolayer graphene membranes were fabricated by removing graphene from its copper growth substrate and transferring to TEM support grids, as described in the Methods section. TMA and TPA were deposited by OMBD directly onto these membranes, and the structure of the resultant films was characterized by TEM imaging and diffraction. Simultaneously, the films were deposited on as-grown Gr–Cu for comparative topographic imaging and film thickness measurements by atomic force microscopy (AFM), see ESI section S1.†

**Fig. 2:**
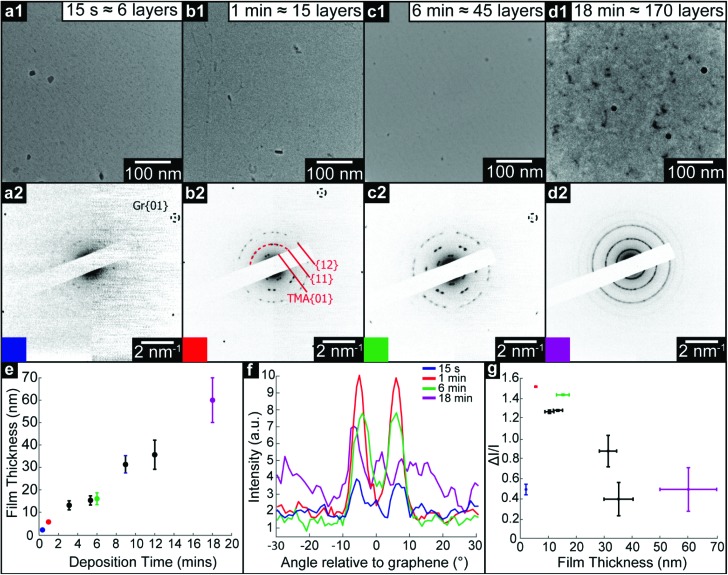
TEM analysis of thin films of TMA deposited onto freestanding graphene. (a1) to (d1), Brightfield TEM images of TMA thin films of increasing deposition time as marked, with corresponding SAED patterns (a2) to (d2) on which graphene and TMA diffraction peaks are labelled. (e) TMA film thickness, as measured by AFM, with deposition time. (f) Azimuthal line profiles of the diffraction intensity through the TMA {11} diffraction peaks, as labelled by the dashed arc on (b2); here 0° is defined by the graphene {01} spots. (g) Modulation of diffraction intensity, Δ*I*/*I*_0_ along TMA {11} azimuths, as a function of film thickness.

Fig. 2(a)–(d) show low-magnification TEM images and corresponding selected area electron diffraction (SAED) patterns of TMA on graphene with increasing deposition time: (a) 15 seconds (measured film thickness of 2.1 ± 0.2 nm, equivalent to ∼6 monolayers, ML), (b) 1 minute (5.5 ± 0.2 nm, ∼15 ML), (c) 6 minutes (16 ± 2 nm, ∼45 ML), and (d) 18 minutes (60 ± 10 nm, ∼170 ML). For all deposition times less than 18 minutes, the TEM images show uniform contrast and the only obvious features can be attributed to residue from the transfer process used to make the graphene membranes, suggesting the TMA is deposited as a uniform thin film, as also confirmed by AFM topography images (see ESI section S1†). For the 18 minutes deposition, there are clear variations in TEM contrast, with features of ∼100 nm, as also seen by AFM (see ESI section 1†), suggesting a granular structure and polycrystalline film.

Despite TEM images showing little contrast, SAED reveals the molecular ordering in TMA layers and their orientation relative to the free-standing graphene substrate. For all films except the 18 minutes deposition, sharp diffraction spots are seen with spacings and (three-fold) symmetry consistent with the 2D chicken-wire TMA structure, as observed by STM, mirrored relative to the graphene lattice (see ESI section 2†). TMA lattice parameters calculated from these diffractions spots are given in Table 1: using the graphene diffraction spots to calibrate the diffraction patterns^43^ allows the TMA lattice parameters to be easily measured from the electron diffractions spots to a significantly higher accuracy and precision than those obtained from STM images.

**Table 1:** Film thicknesses (determined by AFM), lattice parameters and characteristic dose calculated for the monolayer and thin films of TMA. For the 18 min deposition, the angle *γ* is measured from 2D Fourier transforms of acTEM images (see ESI section 9)

Deposition time	Thickness (nm)	*a* (nm)	*b* (nm)	*γ* (°)	Characteristic dose (electrons per nm^2^)
Monolayer (STM)	—	1.65 ± 0.06	1.65 ± 0.06	60 ± 1	—
15 seconds	2.1 ± 0.2	1.64 ± 0.02	1.64 ± 0.02	60.0 ± 0.2	—
1 minute	5.5 ± 0.2	1.64 ± 0.02	1.64 ± 0.02	60.0 ± 0.3	13 ± 3
6 minutes	16 ± 2	1.65 ± 0.02	1.65 ± 0.02	60.0 ± 0.2	100 ± 50

Two distinct orientations of the chicken wire lattice are observed, equally spaced 6.8 ± 0.1° either side of the graphene orientation, indicating an epitaxial relationship between the TMA and graphene lattices. We also observed two orientations of the TMA lattice in STM images of monolayer TMA on Gr–Cu (see ESI section 2†). The STM measured angles of 7 ± 1° relative to the graphene lattice are consistent with the electron diffraction results. Macleod *et al.*^13^ studied supramolecular assembly of monolayer TMA on graphite and graphene by STM, finding similar lattice parameters to those measured here also by STM, and deduced the following epitaxy matrix relating the TMA lattice vectors, 
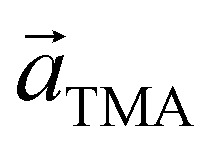
, to those of graphene, 
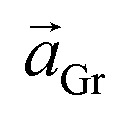
:
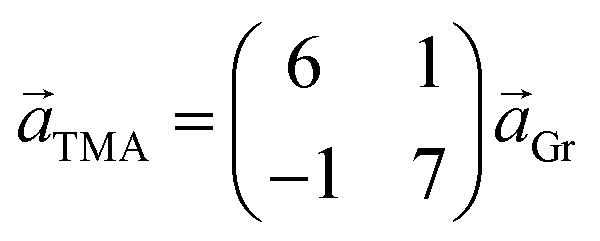


This relationship predicts the TMA lattice parameter to be 
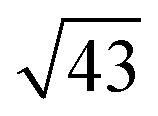
 times the graphene lattice constant, *i.e.*
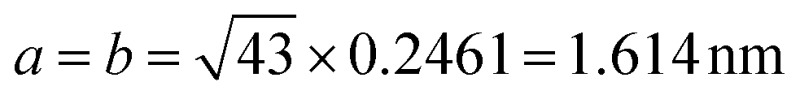
, and the angle between the TMA and the graphene lattice to be 
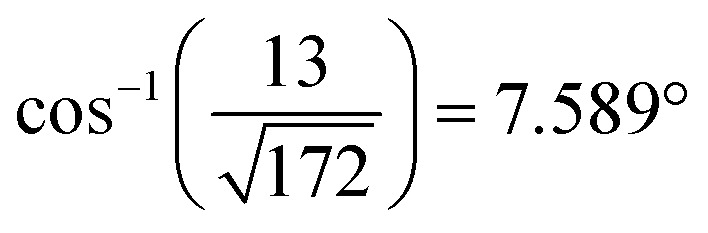
. Although the STM results are consistent, within uncertainties, with these values, the higher accuracy and precision of the SAED measurements reveals that, for the films analyzed in Fig. 2, the relationship between TMA and graphene lattices is not exactly described by such an epitaxy matrix.

This typifies van der Waals epitaxy.^44^ Due to the weak van der Waals interactions between surface and molecular overlayer, the 2D molecular structure is not constrained to exactly follow the lattice parameters of the surface but is relaxed and unstrained, allowing lattice mismatch and differences in symmetry between surface and overlayer. Despite this, the 2D molecular layer is epitaxial (though incommensurate) to the graphene one, in that the orientation of the TMA lattice is defined relative to the graphene lattice. This conclusion is also in agreement with very recent results obtained by analyzing the moiré patterns formed by TMA deposited on HOPG.^45^

For thick films, this epitaxial relationship no longer holds. Electron diffraction from 9 minutes (∼95 ML) and 12 minutes (∼110 ML) films (see ESI section 3†) show many distinct TMA orientations, while the 18 minutes (∼170 ML) TMA deposition shows rings rather than spots, as shown in Fig. 2(d2), though with similar spacings, indicating a polycrystalline film with random in-plane orientation relative to the graphene.

The SAED patterns can be analysed to give a more quantitative insight into the degree of order in the thin films.^28^Fig. 2(f) shows azimuthal line profiles through the {11} TMA diffraction peaks; here 0° is defined by the graphene {01} spots. The two peaks corresponding to the two orientations of TMA are readily apparent for all but the 18 min deposition. Defining *I*_0_ as the average intensity and Δ*I* as the difference between maximum and average intensity, the intensity modulation Δ*I*/*I*_0_ along the arcs gives a relative measure of the order within the film and is plotted in Fig. 2(g) as a function of film thickness. The apparent order increases up to a maximum at ∼20 nm, due to the diffraction peak intensity increasing relative to the local background. Beyond this critical thickness, the intensity modulation (*I*_0_) decays rapidly as more TMA orientations appear, indicating a transition to a rotationally disordered phase with textured but randomly oriented grains.

We note that, as expected, the thin films of TMA rapidly degraded upon exposure to the electron beam. For such materials, structural analysis should be performed with low levels of exposure to the electron beam, below the ‘characteristic dose’.^46^ As described in ESI section 4,† the characteristic dose was calculated for each film by measuring the decay in intensity of diffraction spots with exposure time. All diffraction results were acquired under low dose conditions with total doses less than this characteristic dose, and so are representative of the film structure after assembly.

**Fig. 3:**
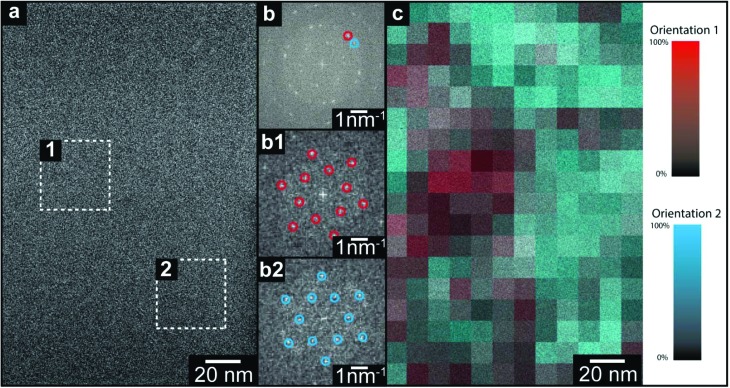
acTEM of TMA (1 minute deposition) on graphene. (a) Brightfield TEM image with corresponding FFTs of selected region 1 (b1) and 2 (b2): spots due to TMA are circled in red/blue showing the two different TMA orientations present. (c) Color map of TMA orientations, formed from processing the image in panel (a); the red intensity corresponds to the intensity of orientation 1 and, correspondingly, the blue intensity is due to orientation 2.

Electron diffraction reveals the spatially averaged crystal structure of TMA on suspended graphene, but leaves important questions open. Are the two orientations within the TMA thin films separated into domains (as suggested by STM in the monolayer), or stacked one on the other? If in domains, what is the domain size? Similarly for the thicker, polycrystalline film, what is the grain size and do they persist through the film thickness? Here we address these questions by directly imaging the TMA layers with acTEM. All images were acquired such that the total exposure was less than the characteristic dose, as measured from the diffraction patterns, to ensure that the observed structure was typical of the as-deposited film (see ESI section 5† for a description of the low-dose acquisition protocol). An example image from a 1 minute TMA deposition on graphene is shown in Fig. 3(a); although there are no immediately obvious features, a 2D fast Fourier transform (FFT, inset in top right corner) shows clear spots and closely resembles the SAED patterns in Fig. 2. This FFT of the whole image is consistent with the expected 2D TMA structure with two orientations, labelled by red and blue circles in the FFT (henceforth orientation 1 and 2). Selecting smaller areas of the image, FFTs corresponding to only one orientation are found, as shown in Fig. 3(b1) and (b2) taken from the dashed boxes 1 and 2 in Fig. 3(a). By analyzing the relative intensity of these two orientations in selected area FFTs (see ESI section 6†), a map of the local TMA orientation can be constructed, as shown in Fig. 3(c). Here, the intensity of red gives the intensity of orientation 1 and, correspondingly, the intensity of blue gives that of orientation 2. This color map thus shows that the two orientations are distinct – *i.e.* they are separated into domains, with stacked layers of the same orientation in each domain – and reveals that the average domain size is ∼40 nm.

**Fig. 4:**
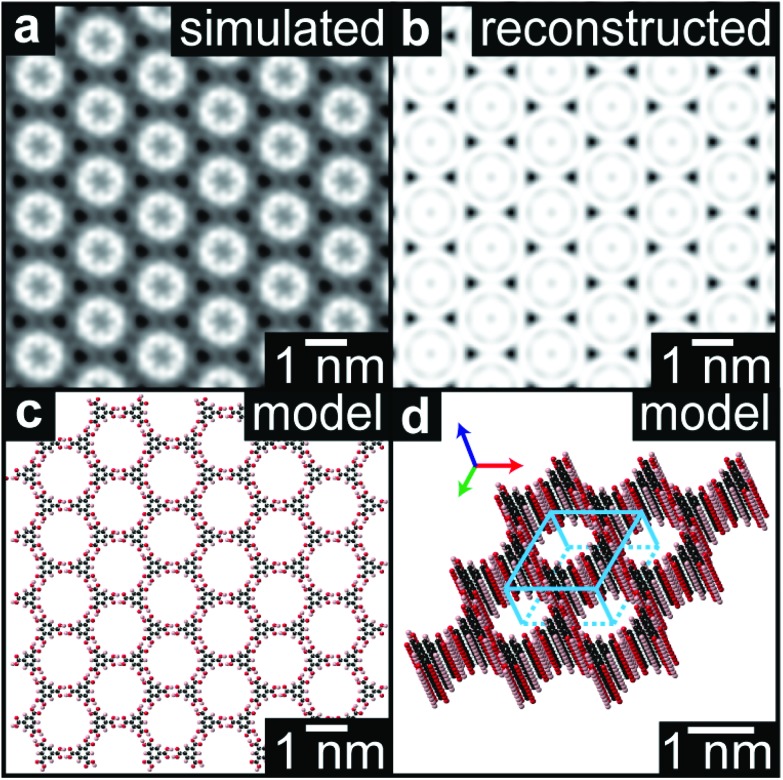
High resolution reconstruction of the TMA film structure. (a) Reconstructed high-resolution image of the TMA structure from the acTEM image data in Fig. 3. (b) Multislice image simulation from the molecular model shown in plan view in (c) and perspective view in (d).

Unlike the diffraction patterns, the Fourier transform operation generates both amplitude and phase information which, when recombined, can be used to reconstruct a real space image. Fig. 4(b) is a reconstructed TEM image of the TMA film (1 minute deposition), taking the amplitude and phase from the peaks in the FFT out to 7 nm^–1^ (for further details see ESI section 8†). This image is consistent with a multislice image simulation,^47^Fig. 4(a) assuming a stacked molecular structure, as shown in the molecular models in Fig. 4(c) and (d), but not with structures that do not assume direct molecular stacking (see ESI section 7†). acTEM imaging thus proves that the TMA molecules are stacked vertically one on another, consistent with density function theory (DFT) calculations of the most energetically favorable stacking geometry,^48^ and hence that initially TMA film growth proceeds *via* a layer-by-layer, or Frank–van der Merwe, growth mode.^49^ Significantly, this stacking is expected to create well-ordered arrays of high-aspect ratio nanopores, around 1.5 nm in diameter and up to 20 nm deep, open at the top and reaching the pristine graphene surface at the bottom.

For films beyond the critical thickness, where SAED shows polycrystalline rings, high-resolution imaging shows a small grain size (<30 nm) with evidence that the grains do not normally persist through the film (see ESI section 9†), again indicative of a polycrystalline film.

### Structural transition in TPA thin-films

**Fig. 5:**
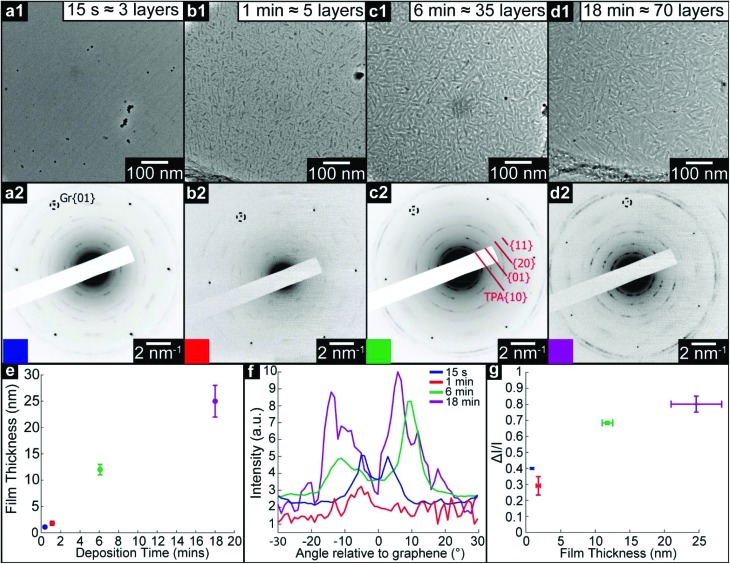
TEM analysis of thin films of TPA on graphene. (a1) to (d1), Brightfield TEM images of TPA thin films of increasing deposition time (15 s, 1 min, 6 min, and 18 min), with corresponding electron diffraction patterns (a2) to (d2) on which graphene and TPA diffraction peaks are labelled. (e) TPA film thickness, as measured by AFM, as a function of deposition time. (f) Azimuthal line profiles of the diffraction intensity through the TPA {01} diffraction peaks; 0° is defined by the graphene {10} spots. (g) Modulation of diffraction intensity, Δ*I*/*I*_0_ along TPA {11} azimuths, as a function of film thickness.

We used a similar methodology to study structural transitions in TPA thin films on graphene. Fig. 5(a)–(d) show brightfield TEM images and corresponding SAED patterns of TPA thin films with increasing deposition times as marked. The film thickness was again measured by AFM (see ESI section 1†). The TEM images show a strikingly different trend to that observed for TMA: fiber-like features ∼100 nm in length are apparent from 1 minute and persist at longer deposition times, suggesting that TPA forms 3D islands from an early stage. These features are also clearly visible in AFM images (see ESI section 1†). Such topographical changes, combined with STM evidence for an initial wetting monolayer, suggest that TPA on graphene is following a layer-plus-island, or Stranski–Krastanov, growth mode.^49^

The corresponding SAED patterns also show behavior distinct to that observed for TMA. For the 15 s deposition, clear diffraction spots are apparent which are consistent with the brickwork 2D lattice observed for the monolayer by STM, with 6 distinct orientations symmetrically arranged relative to the graphene lattice (see ESI section 10†). As the deposition time increases, although the graphene diffraction spots are still as clear and well-defined as before, SAED from the TPA thin films gives short arcs rather than sharp spots. Azimuthal line profiles through the TPA {01} arcs are shown in Fig. 5(f) with the corresponding Δ*I*/*I* shown in Fig. 5(g). The line profiles are roughly symmetric relative to the graphene {01}, indicating that van der Waals epitaxy still plays an important role in defining the growth orientations. Both the width of the TPA {01} diffraction peaks and Δ*I*/*I* increase with deposition time. However, careful analysis of the positions of these diffraction arcs shows an important difference compared to TMA: the electron diffraction spacings, and hence the 2D projection of the lattice parameters, change with deposition time. This is also observed in FFTs of high-resolution images (see ESI section 11†) which show the same lattice parameters as the corresponding diffraction patterns. Interestingly, these FFTs show distinct spots rather than arcs, indicating that the SAED diffraction arcs are due to small changes in orientation between grains rather than molecular tilting within grains.^50^ The sharp spots in the FFTs enable accurate measurements of the angle between lattice vectors, as presented in Table 2. The high-resolution images also show that the crystalline grain size here is ∼20 nm (see ESI section 7†), consistent with the width of the fibers in the low magnification brightfield images.

**Table 2:** Film thickness, lattice parameters and characteristic dose for TPA on graphene. Also shown are the lattice projections looking down the *c*-axis of the reported TPA bulk structure.^42^ Note that the exact 3D crystallographic orientation of the thicker films here is not known

Deposition time	Thickness (nm)	*a* (nm)	*b* (nm)	*γ* (°)	Unit cell area (nm^2^)
Monolayer (STM)	—	0.95 ± 0.02	0.75 ± 0.06	53 ± 3	0.57 ± 0.05
15 seconds	1.1 ± 0.4	0.95 ± 0.02	0.74 ± 0.02	50 ± 2	0.54 ± 0.02
1 minute	1.2 ± 0.2	0.90 ± 0.02	0.72 ± 0.02	53 ± 2	0.52 ± 0.02
6 minutes	12 ± 3	0.85 ± 0.02	0.62 ± 0.02	57 ± 2	0.44 ± 0.02
18 minutes	25 ± 1	0.86 ± 0.02	0.60 ± 0.02	56 ± 2	0.43 ± 0.02
Bulk projection	—	0.92 ± 0.01	0.65 ± 0.01	52 ± 1	0.47 ± 0.01

The key result from TEM analysis of TPA films on graphene is the change in projected lattice parameters with deposition time, as summarized in Table 2. The reduction in both *a* and *b* lattice parameters, and the subsequent contraction of the unit cell area, is consistent with the molecules tilting with respect to the graphene surface and hence packing more densely, as in the bulk structure. The gradual change observed here reflects the smooth transition that can occur from the 2D, flat, structure to the 3D, tilted, structure.

The differences between the TMA and TPA film deposition are intriguing. TMA deposition results in layer-by-layer growth, templating the 2D structure upwards and creating open nanopores up to ∼20 nm deep and ∼1.5 nm wide, until, after a critical thickness of >20 nm, the film abruptly becomes polycrystalline with random in-plane orientations. By contrast, TPA rapidly forms fiber-like islands after the first 2D molecular overlayer and its lattice parameters gradually reduce from those of the 2D structure, smoothly becoming more consistent with the bulk structure.

We speculate on the origin of these differences through inspection of their 2D structure relative to their 3D crystallography. Energetically, the 2D structure is stabilized by interactions with the surface whilst the 3D crystallography is determined only by the intermolecular interactions; although the dominant forces driving the transition from 2D to 3D are not obvious and are worthy of future study, the differences in 3D structure between TMA and TPA give insight into their contrasting behaviour. The TPA bulk structure is characterized by parallel hydrogen-bonded lamellar rows and its (001) plane displays a structural similarity with the 2D lattice of TPA-on-graphene (the main difference being a contracted lattice parameter in 3D, through tilting of the molecule towards the [223] direction). As the film thickness increases, surface-interactions become less significant and the intermolecular interactions are expected to increasingly dominate. This explains the observed behavior of TPA; the 2D structure is a distorted (strained) component of the 3D structure and hence a smooth transition can occur. This also explains the formation of crystallites (here fiber-like) to reduce strain.^51^

However, the 3D crystal structure of TMA is composed by interpenetrating non-planar chicken-wire frameworks and is thus very different and, crucially, topologically distinct from the planar 2D molecular lattice of the monolayer. As a result, the 2D layer cannot be thought of as a strained component of the 3D structure, and no smooth transition is possible. Hence, the TMA templates from the initial 2D layer until an abrupt transition to a polycrystalline phase; the 2D structure is topologically protected against transitions to the 3D structure.

## Conclusions

We demonstrate fundamentally new insight into the growth of supramolecular thin films on surfaces through a detailed study into the structural evolution of layers of prototypical benzenecarboxylic acids. To achieve this, we have used an innovative combination of low-dose acTEM and STM to accurately determine molecular-resolution structural information on films of increasing thickness from monolayer through to tens of nanometers, a precision and range that is difficult to attain by other analytical techniques. Although this approach will not be applicable to all supramolecular assembly problems as it requires atomically thin substrates and comparatively stable molecular assemblies for acTEM, it has the potential to provide sub-nanometre resolution structural information on complex molecular thin films, as demonstrated here for the prototypical systems of TMA and TPA on graphene. For both, the structure and orientation of the first molecular overlayer are dictated by the comparatively strong hydrogen bonding between molecules and the interactions with the graphene surface that determine a weak van der Waals epitaxial relationship. As the film thickness increases beyond a monolayer, however, TMA and TPA display distinctly different behaviors, despite their chemical similarities. TMA templates from the 2D structure, stacking molecular layers directly on top of each other until, above a certain thickness, the film transitions to a polycrystalline phase with random in-plane orientations. By contrast, TPA forms fiber-like islands and the in-plane lattice parameters change continuously with thickness, smoothly becoming more consistent with the bulk structure. We propose that these differences in behavior can be understood through comparison between the 2D and 3D structures of the two molecules: the bulk structure of TMA is topologically distinct from the monolayer structure with no possible smooth transition between the two, whereas, for TPA, tilting of the molecules with respect to the surface gives a continuous transition from 2D to 3D structures. As a result, the 2D TMA structure is topologically protected and templates through the initial film growth. This new concept of topological protection of the 2D monolayer structure is expected to play an important role in the design of functional thin films by controlled supramolecular assembly.

## Experimental details

### Graphene growth

Graphene was grown on low cost copper foils *via* low pressure CVD using methane as a feedstock.^52^ First, the copper foils were electropolished in a solution containing orthophosphoric acid and urea (5 V, 1.5 A).^53^ After rinsing off the electrolyte with deionized water and isopropanol, the polished foils were sonicated in acetone, and then rinsed again with isopropanol and dried with nitrogen. Afterwards they were loaded into a quartz tube in a tube furnace, which was pumped to vacuum below 1 × 10^–3^ mbar. Hydrogen was flowed at 10 standard cubic centimeters per minute (sccm), raising the pressure to 1 × 10^–2^ mbar. The furnace was heated to 1000 °C, and left to anneal for 20 min. Methane was then added at 3 sccm for 30 min. This yields copper foils that are >99% covered with predominantly single layer graphene of high-quality.^54^ Under these growth conditions, the typical graphene grain size is found to be ∼20 μm.

### Graphene TEM grid fabrication

To transfer graphene to TEM grids, the graphene-coated foils were first spin-coated with formvar (3.4 mg mL^–1^) using spin speed 3000 rpm, ramp 0.1 s, and dwell 45 s. The coated foils were then placed into ammonia persulphate to etch away the copper overnight. Once the copper was removed, the foils were transferred to five successive beakers of deionized water, to remove any remaining etchant. The floating stack was then scooped using SiN TEM supports (from Silson) and left to dry in air. The grids were then placed in chloroform for 10 min to remove the formvar. They were then transferred to acetone, and then to a critical point dryer, to dry without surface tension breaking the films. Finally, the TEM grids were further cleaned by heating on a hotplate at 200 °C for 2 h.

### Molecular deposition

TPA or TMA molecules (Sigma Aldrich: TMA 1,3,5 benzenetricarboxylic acid, 95% purity, CAS 554-95-0 and TPA teraphtalic acid, 98% purity, CAS 100-21-0) were deposited by OMBD in a chamber with a base pressure <10^–5^ mbar, with source deposition temperatures of ∼265 °C and ∼230 °C for TMA and TPA, respectively, with the substrates at ambient temperature. The deposition rate was monitored by quartz microbalance, and the film thickness was measured by AFM after deposition (see below and ESI section 1†). Molecules were deposited simultaneously on different substrates: graphene-on-TEM grids, graphene-on-copper, and HOPG.

### Scanning tunnelling microscopy

STM images were acquired under ambient conditions at the liquid–solid interface under a drop of heptanoic acid with a Veeco STM with Nanoscope E controller and an A-type scanner, using mechanically-sheared Pt/Ir (90/10) tip. Typical tunneling parameters were 80 pA and –1 to –1.5 V for molecular imaging and 800 pA and –0.1 V for atomic resolution imaging of the underlying graphene. Negative bias, here applied to the sample, corresponds to filled state imaging. STM images were drift-corrected by using the graphene atomic lattice as a reference. All STM images were processed using the WSxM software.^55^

### Atomic force microscopy

An Asylum Research MFP3D-SA was used in AC-mode (or tapping mode) for topographic imaging and combined AC-mode and contact mode for thickness measurements, as described in ESI section 1.†

### Transmission electron microscopy

For electron diffraction, a JEOL 2100 TEM was used, operating at 200 kV: the selected area aperture acquired signal from circular areas 3 μm in diameter. (Note that concomitant with the ∼20 μm graphene grain size, only one graphene orientation is present in each TEM image or diffraction pattern presented here.) All angles measured from the SAED patterns were averaged across all relevant diffraction peaks and the uncertainties calculated from the standard error of these values. The dose was estimated by measuring the current draining to earth from the phosphor screen when illuminated by the electron beam. This was adjusted to ≈5 e^–^ Å^–2^ s^–1^ for diffraction. For acTEM, a JEOL ARM 200F was used, operating at 80 kV, with CEOS probe and image aberration correction. Again, the dose was measured through the screen and was adjusted to ≈100 e^–^ Å^–2^ s^–1^ for high resolution imaging.

## Supplementary Material

Supplementary information

## References

[cit1] Slater A. G., Beton P. H., Champness N. R. (2011). Chem. Sci..

[cit2] Elemans J. A. A. W., Lei S., De Feyter S. (2009). Angew. Chem., Int. Ed..

[cit3] Barth J. V. (2007). Annu. Rev. Phys. Chem..

[cit4] Theobald J. A., Oxtoby N. S., Phillips M. A., Champness N. R., Beton P. H. (2003). Nature.

[cit5] Stepanow S., Lingenfelder M., Dmitriev A., Spillmann H., Delvigne E., Lin N., Deng X., Cai C., Barth J. V., Kern K. (2004). Nat. Mater..

[cit6] Liang H., He Y., Ye Y., Xu X., Cheng F., Sun W., Shao X., Wang Y., Li J., Wu K. (2009). Coord. Chem. Rev..

[cit7] Kudernac T., Lei S., Elemans J. A. A. W., De Feyter S. (2009). Chem. Soc. Rev..

[cit8] Yang J., Yan D., Jones T. S. (2015). Chem. Rev..

[cit9] Grumelli D., Wurster B., Stepanow S., Kern K. (2013). Nat. Commun..

[cit10] Copéret C., Chabanas M., Petroff Saint-Arroman R., Basset J.-M. (2003). Angew. Chem., Int. Ed..

[cit11] Otero R., Gallego J. M., De Parga A. L. V., Martín N., Miranda R. (2011). Adv. Mater..

[cit12] Pollard A. J., Perkins E. W., Smith N. A., Saywell A., Goretzki G., Phillips A. G., Argent S. P., Sachdev H., Müller F., Hüfner S., Gsell S., Fischer M., Schreck M., Osterwalder J., Greber T., Berner S., Champness N. R., Beton P. H. (2010). Angew. Chem., Int. Ed..

[cit13] MacLeod J. M., Lipton-Duffin J. A., Cui D., De Feyter S., Rosei F. (2015). Langmuir.

[cit14] Macleod J. M., Rosei F. (2014). Small.

[cit15] Korolkov V. V., Svatek S. A., Summerfield A., Kerfoot J., Yang L., Taniguchi T., Watanabe K., Champness N. R., Besley N. A., Beton P. H. (2015). ACS Nano.

[cit16] Zhang Z. X., Huang H. L., Yang X. M., Zang L. (2011). J. Phys. Chem. Lett..

[cit17] Georgakilas V., Otyepka M., Bourlinos A. B., Chandra V., Kim N., Kemp K. C., Hobza P., Zboril R., Kim K. S. (2012). Chem. Rev..

[cit18] Chen W., Chen S., Qi D. C., Gao X. Y., Wee A. T. S. (2007). J. Am. Chem. Soc..

[cit19] Coletti C., Riedl C., Lee D. S., Krauss B., Patthey L., von Klitzing K., Smet J. H., Starke U. (2010). Phys. Rev. B: Condens. Matter.

[cit20] Dong X., Shi Y., Zhao Y., Chen D., Ye J., Yao Y., Gao F., Ni Z., Yu T., Shen Z., Huang Y., Chen P., Li L. J. (2009). Phys. Rev. Lett..

[cit21] Kozlov S. M., Viñes F., Görling A. (2011). Adv. Mater..

[cit22] Jo G., Choe M., Lee S., Park W., Kahng Y. H., Lee T. (2012). Nanotechnology.

[cit23] Marsden A. J., Rochford L. A., Wood D., Ramadan A. J., Laker Z. P. L., Jones T. S., Wilson N. R. (2016). Adv. Funct. Mater..

[cit24] Lee W. H., Park J., Sim S. H., Lim S., Kim K. S., Hong B. H., Cho K. (2011). J. Am. Chem. Soc..

[cit25] Huang H., Huang Y., Wang S., Zhu M., Xie H., Zhang L., Zheng X., Xie Q., Niu D., Gao Y. (2016). Crystals.

[cit26] Strong L., Whitesides G. M. (1988). Langmuir.

[cit27] Bonnerot A., Chollet P. A., Frisby H., Hoclet M. (1985). Chem. Phys..

[cit28] Cheng M., Ho J. T., Hui S. W., Pindak R. (1987). Phys. Rev. Lett..

[cit29] Pope C. R., Unger V. M. (2012). Curr. Opin. Struct. Biol..

[cit30] Ubarretxena-Belandia I., Stokes D. L. (2012). Curr. Opin. Struct. Biol..

[cit31] Nogales E. (2015). Nat. Methods.

[cit32] Pantelic R. S., Meyer J. C., Kaiser U., Stahlberg H. (2012). Solid State Commun..

[cit33] Lackinger M., Griessl S., Heckl W. M., Hietschold M., Flynn G. W. (2005). Langmuir.

[cit34] Griessl S., Lackinger M., Edelwirth M., Hietschold M., Heckl W. M. (2002). Single Mol..

[cit35] Korolkov V. V., Allen S., Roberts C. J., Tendler S. J. B. (2012). J. Phys. Chem. C.

[cit36] Shayeganfar F., Rochefort A. (2014). Langmuir.

[cit37] Addou R., Batzill M. (2013). Langmuir.

[cit38] Zhang W., Nefedov A., Naboka M., Cao L., Wöll C. (2012). Phys. Chem. Chem. Phys..

[cit39] Banerjee K., Kumar A., Canova F. F., Kezilebieke S., Foster A. S., Liljeroth P. (2016). J. Phys. Chem. C.

[cit40] Zhou Q., Li Y., Li Q., Wang Y., Yang Y., Fang Y., Wang C. (2014). Nanoscale.

[cit41] Duchamp D. J., Marsh R. E. (1969). Acta Crystallogr., Sect. B: Struct. Crystallogr. Cryst. Chem..

[cit42] Bailey M., Brown C. J. (1967). Acta Crystallogr..

[cit43] Wilson N. R., Pandey P., Beanland R., Young R. J., Kinloch I., Gong L., Liu Z., Suenaga K., Rourke J. P., York S. J., Sloan J. (2009). ACS Nano.

[cit44] Utama M. I. B., Zhang Q., Zhang J., Yuan Y., Belarre F. J., Arbiol J., Xiong Q. (2013). Nanoscale.

[cit45] Spitzer S., Helmle O., Ochs O., Horsley J., Martsinovich N., Heckl W. M., Lackinger M. Faraday Discuss..

[cit46] Egerton R. F. (2013). Ultramicroscopy.

[cit47] Ishizuka K., Uyeda N. (1977). Acta Crystallogr., Sect. A: Cryst. Phys., Diffr., Theor. Gen. Cryst..

[cit48] Shayeganfar F. (2014). J. Phys.: Condens. Matter.

[cit49] Ratsch C., Venables J. A. (2003). J. Vac. Sci. Technol., A.

[cit50] Hui S. (1989). J. Electron Microsc. Tech..

[cit51] Käfer D., Ruppel L., Witte G. (2007). Phys. Rev. B: Condens. Matter.

[cit52] Li X., Cai W., An J., Kim S., Nah J., Yang D., Piner R., Velamakanni A., Jung I., Tutuc E., Banerjee S. K., Colombo L., Ruoff R. S. (2009). Science.

[cit53] Zhang B., Lee W. H., Piner R., Kholmanov I., Wu Y., Li H., Ji H., Ruoff R. S. (2012). ACS Nano.

[cit54] Wilson N. R., Marsden A. J., Saghir M., Bromley C. J., Schaub R., Costantini G., White T. W., Partridge C., Barinov A., Dudin P., Sanchez A. M., Mudd J. J., Walker M., Bell G. R. (2013). Nano Res..

[cit55] Horcas I., Fernández R., Gómez-Rodríguez J. M., Colchero J., Gómez-Herrero J., Baro A. M. (2007). Rev. Sci. Instrum..

