# Supplementary material for: Monolayer-to-thin-film transition in supramolecular assemblies: the role of topological protection
Source: Nanoscale. 2017 Aug 1;9(33):11959–68. doi: 10.1039/c7nr03588h (PMC5778949; doi:10.1039/c7nr03588h)
Supplement: Supplementary file 1 [file NR-009-C7NR03588H-s001.pdf]

Electronic Supplementary Material (ESI) for Nanoscale.
This journal is © The Royal Society of Chemistry 2017

Electronic Supplementary Information for:

Monolayer-to-thin-film transition in supramolecular assemblies: the role of topological protection

Zachary P. L. Laker, Alexander J. Marsden, Oreste De Luca, Ada Della Pia, Luís M. Alves Perdigão, Giovanni Costantini, Neil R. Wilson

Contents

S1: Atomic force microscopy (AFM) of TMA and TPA thin films on graphene 2
S2: Two orientations of TMA on graphene observed by STM. 4
S3: Film thickness dependence of diffraction from TMA on graphene. 7
S4: Measuring the characteristic dose of TMA on graphene 8
S5: Low dose acTEM image acquisition protocol.. 11
S6: Mapping the orientation of domains in the TMA thin film.. 12
S7: Mapping the orientation of grains in the TPA thin films 14
S8: Multislice acTEM image simulations of TMA thin films... 15
S9: acTEM imaging of polycrystalline nature of thicker TMA films 19
S10: Demonstration of the 6 orientations of TPA apparent by SAED 21
S11: acTEM imaging of TPA thin films.. 22
References... 24

***S1**: Atomic force microscopy (AFM) of TMA and TPA thin films on graphene*

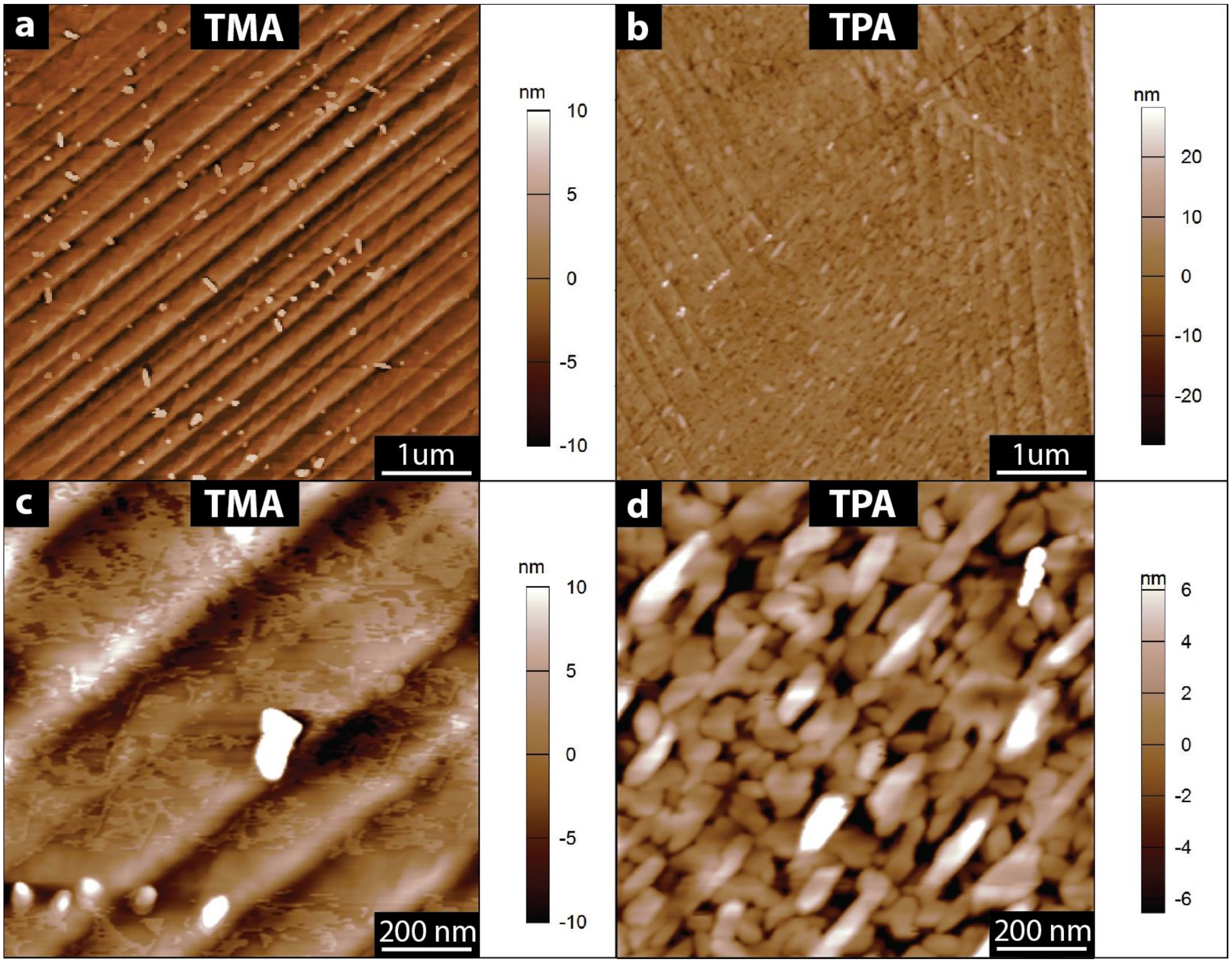

Figure S1a. Tapping mode AFM images of TMA, (a) and (c), and TPA films, (b) and (d), on graphene on copper for 6 minute depositions.

The topography of TMA and TPA films was studied using AFM. Figure S1a shows tapping mode images of 6 minute deposition films for TMA (a,c) and TPA (c,d). The TMA and TPA films themselves appear in stark contrast to each other; TMA shows a flat, continuous thin-film whereas TPA appears to grow in small lozenge-shaped crystallites. This is in agreement with the structures studied using TEM.

The thicknesses of TMA and TPA films were also measured using AFM. Figure S1b shows measurements of film thickness for the TMA and TPA depositions onto graphene on copper. Panel (a) is a tapping mode AFM topography image of an 18 minute-deposition film of TPA on graphene on copper after a trench has been scratched through the film. The trench is scratched by repeatedly scanning a small region in contact mode. Panel (b) is an averaged line-profile from the white dashed rectangle in (a), from which the film thickness was measured.

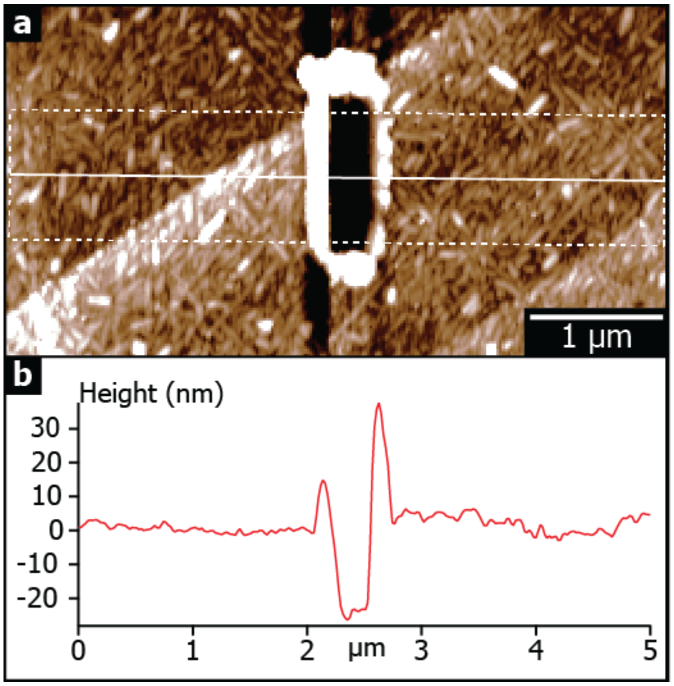

Figure S1b. AFM film thickness measurement. Panel (a) shows an AFM image of an 18-minute deposition of TPA on graphene on copper, after a trench has been scratched using contact mode. Panel (b) is an average line profile, from the dashed rectangle in (a), from which the film thickness is measured.

S2: *Two orientations of TMA on graphene observed by SAED and STM.*

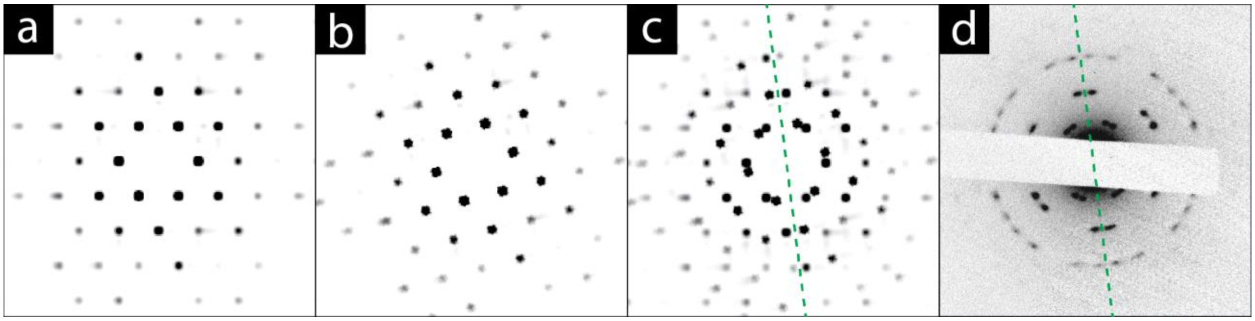

Figure S2a. SAED simulations of TMA on graphene. (a) Simulated SAED of a TMA monolayer. (b) Simulated SAED of a TMA monolayer rotated 13.6º relative to (a). (c) Addition of SAED simulations presented in (a) and (b) showing close similarity to experimental SAED image presented in (d). The green dashed line in (c) and (d) shows the orientation of graphene reflections which the two TMA grains are oriented with respect to. Simulations were performed using clTEM, an open-source multislice simulation package (M. A. Dyson, clTEM: OpenCL TEM/STEM simulation code, URL ⟨http://github. com/ADyson/clTEM⟩, 2014).

Analysis of SAED simulation patterns from TMA reveals how two distinct orientations of the chicken-wire superstructure arise in the experimental SAED patterns through addition of individual SAED patterns from separate TMA grains. Combining individual SAED simulation patterns of TMA monolayers rotated 13.6º relative with respect to each other reveals a SAED pattern similar to the experimental images.

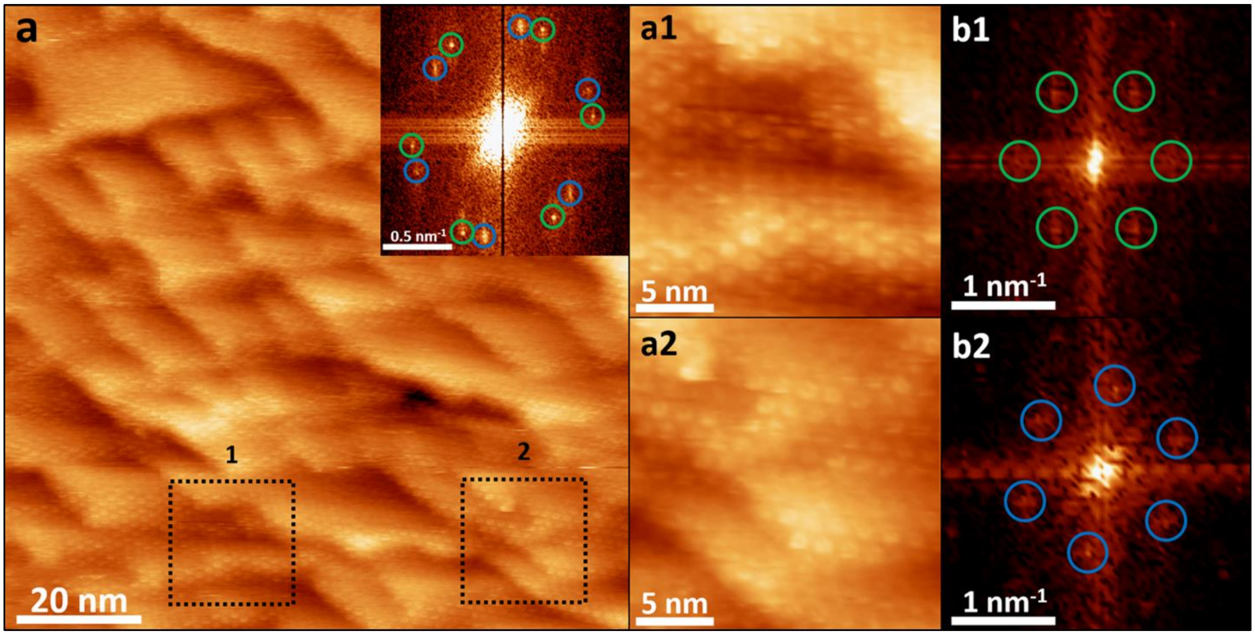

Figure S2b. STM of TMA on graphene. (a) Large scale STM image (100×100 nm^2) of a TMA monolayer at the interface between heptanoic acid and graphene on copper. Tunneling parameters: V = −1.3 V, I = 50 pA. Inset: FFT of the whole image indicating the presence of two distinct orientations of the TMA chicken-wire lattice. (a1) and (a2) are magnified views of the regions in (a) labelled as 1 and 2, respectively. (b1) and (b2) are the corresponding FFTs. (a1) and (a2) images have been drift-corrected by using atomic resolution images of the graphene substrate.

Analysis of STM images from TMA on Gr-Cu shows two distinct orientations of the chicken-wire superstructure with respect to the underlying graphene lattice. Figure S2b (a) shows a large-scale STM image with TMA molecules completely covering the graphene surface and the inset displays its fast Fourier transform (FFT). Two different rotational domains (relative angle of 14° ± 2°) can be identified in the FFT for the TMA chicken-wire structure, and are indicated by green and blue circles, respectively. Figures S2b (a1) and (a2) show magnified images of the two distinct sub-regions marked by dotted squares in figure S2b (a). The corresponding FFTs are

shown in figures S2b (b1) and (b2), respectively, and display only one orientation each. STM images were drift-corrected by using the graphene atomic lattice as a reference. In particular, the acquisition of STM images with both molecular and substrate atomic resolution allows the images to be rescaled based on the known lattice periodicity of graphene. Once the correct unit cell of the molecular assembly has been determined, other images showing only molecular resolution can be rescaled accordingly.

S3: *Film thickness dependence of diffraction from TMA on graphene.*

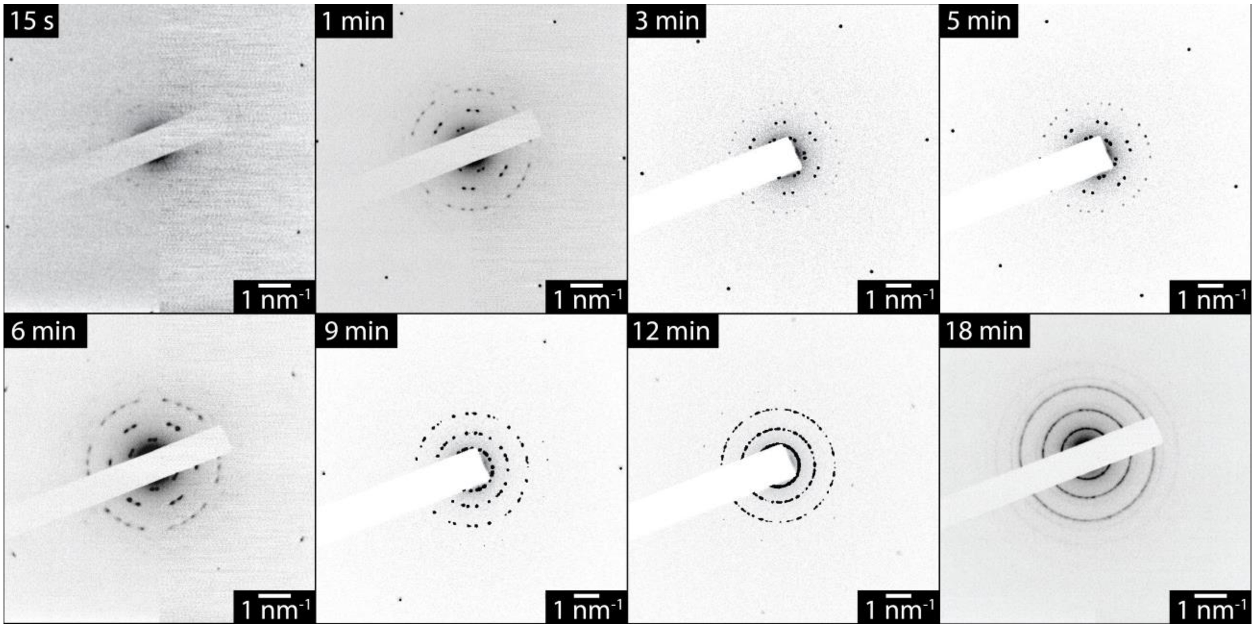

Figure S3. Electron diffraction patterns from TMA thin films of increasing thickness on graphene. Two distinct orientations are visible until 6 min; longer depositions show increasing amounts of orientational disorder.

$\boldsymbol{S4}$: *Measuring the characteristic dose of TMA on graphene*

The use of electron microscopy to study organic thin films is restricted by the lifetime of the organic molecules, and their supramolecular structure, under the electron beam. To quantify this lifetime, diffraction patterns from organic thin films can be analyzed to find the characteristic dose D_C – defined as the dose after which the diffraction spot intensity has been reduced by a factor $1/e$, and after which the structure is believed to have been significantly damaged:[1]

$$D_c = Dt_c$$

where D is the electron dosing rate of the electron beam at the sample plane and t_c is the time after which the diffraction spot intensity has been reduced by a factor $1/e$ (characteristic time).

Figure S4 (a) to (d) show four images from a sequence of diffraction patterns that were recorded continuously over 38 seconds, with a diffraction pattern acquired every 0.3 seconds, from a 1 minute-deposition of TMA on graphene. Throughout the exposure, the dosing rate was kept constant at (1.3±0.3) $e^{-}Å^{-2}s^{-1}$. The spots attributed to TMA are clear, as are those from the graphene substrate. From the series it is apparent that the TMA diffraction spots reduce in intensity with increasing exposure time, and are not visible after 33 seconds, whilst the graphene spots remain constant.

The intensity in a diffraction peak can be quantified by summing the intensity in a small area around its maximum, and then subtracting this value by the average background intensity summed over an equivalent area. This is repeated for each image in the sequence. Three diffraction peaks for the TMA are chosen, one from each order of the diffraction pattern, and one diffraction peak for the graphene. The resulting intensities are plotted against exposure time in Figure S4 (e).

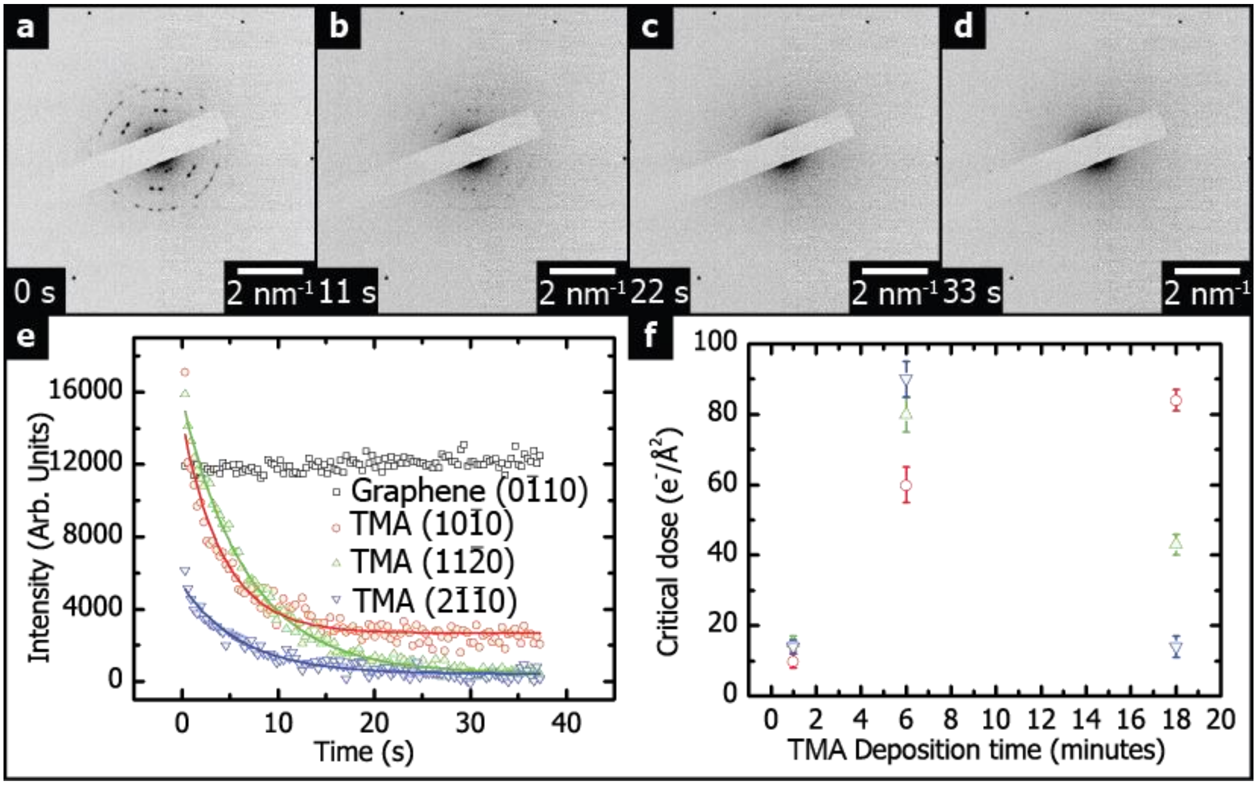

Figure S4. Measuring the critical electron dose for TMA thin films. (a)-(d) show diffraction patterns from a series taken from the same area with increasing exposure time. The time of each image is indicated in the bottom left corner. Plotting the intensity of the diffraction spots, (e), shows exponential decays, caused by the damage from the electron beam. The characteristic dose of the decay is plotted for the 1, 6 and 18 minute-depositions in (f).

For the graphene diffraction spot, the intensity remains constant throughout the exposure, suggesting only negligible damage to the graphene lattice. On the other hand, for all the TMA diffraction peaks there is an exponential decrease in intensity. This demonstrates a disruption to the crystal structure of the TMA layer. After a long exposure, there are no diffraction spots, consistent with a disordered material. The weak bonding, poor conductance, and large population of light atoms present in organic molecular systems such as TMA result in rapid damage when subject to the TEM beam. High energy electrons act to break bonds between atoms as well as

intermolecular bonds, leaving collections of atoms and free radicals which can desorb from the crystal or attack neighboring molecules, resulting in further damage. These areas rapidly become amorphous, losing all crystallinity, and no longer contribute to the diffraction reflections for the remaining crystal. The damaged areas continue to grow, with undamaged crystalline areas decaying in proportion, until the molecular crystal is fully amorphous.[1–3]

S5: *Low dose acTEM image acquisition protocol*

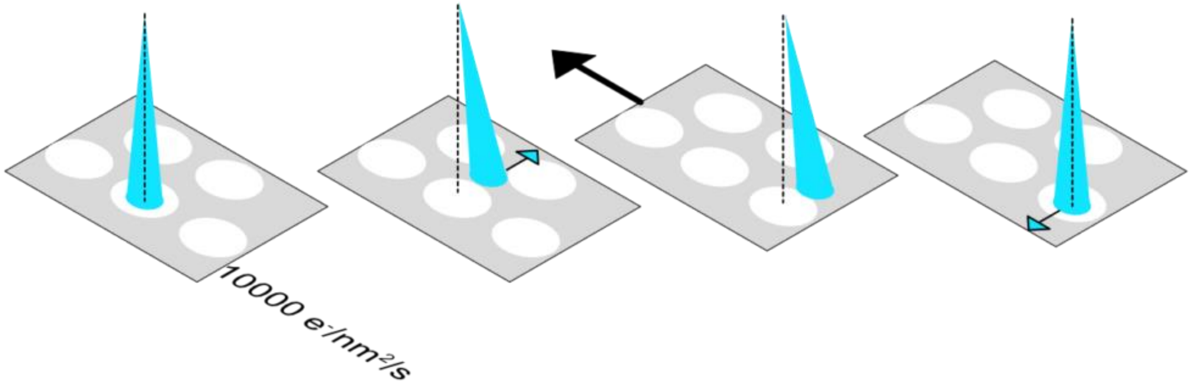

Figure S5. Schematic of the low dose acquisition procedure.

The low dose image acquisition protocol implemented for this work takes advantage of the regularly spaced holes in the TEM supports, and of the fact that the molecular film covers the holes uniformly. First, the microscope conditions (focus and astigmatism) are set using part of the film that has already been damaged by the electron beam. The beam is then shifted away from the sample and the stage moved by a known distance to another hole. After that, and allowing time for the stage drift to settle, the beam is shifted back and images are captured instantly.

S6: *Mapping the orientation of domains in the TMA thin film*

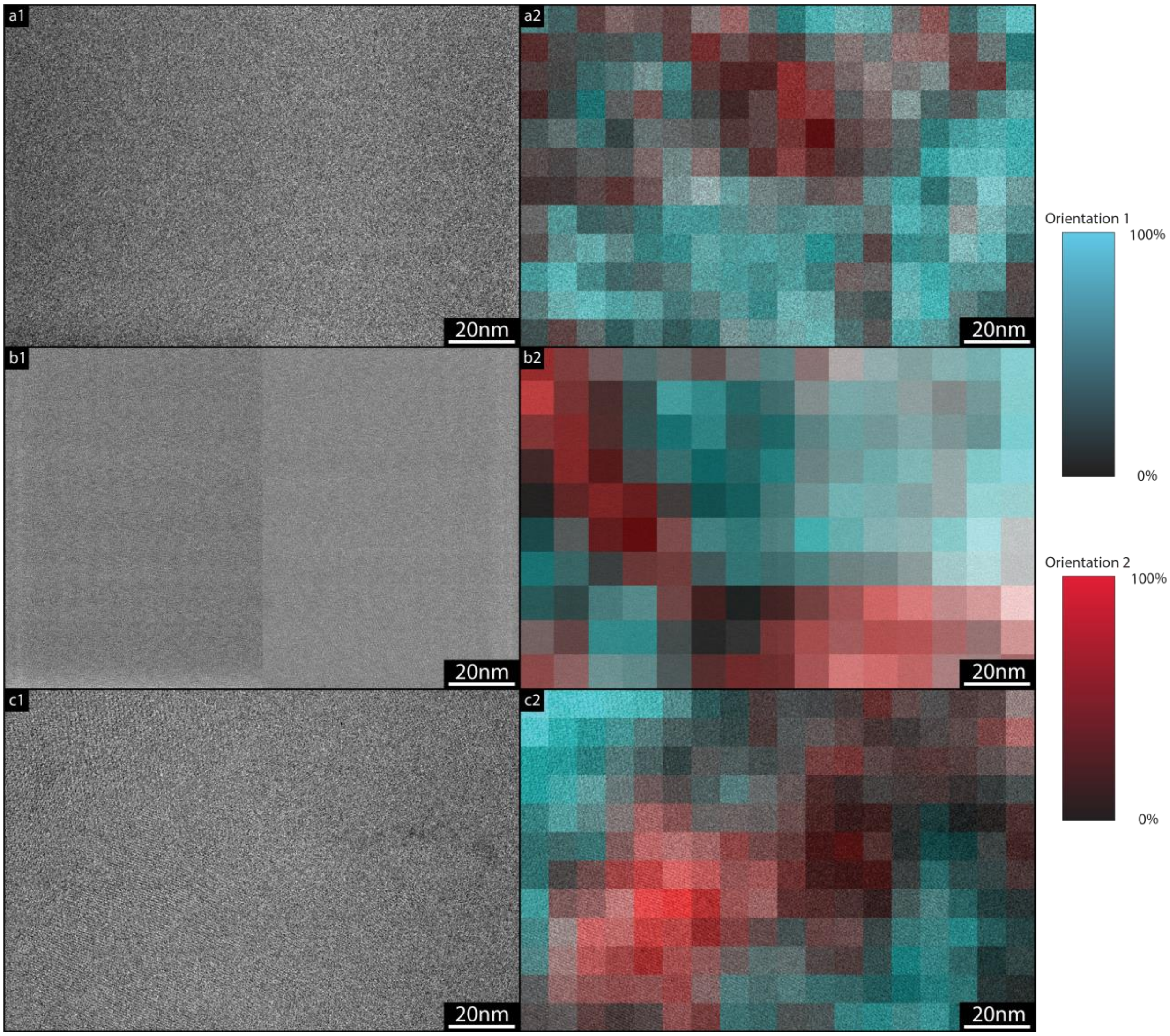

Figure S6. acTEM grain orientation maps for different thickness TMA. Red/Cyan 'pixels' are overlaid on the original acTEM image to indicate regions with one preferential grain orientation. (a1, a2) 1 minute deposition without/with grain overlay. (b1, b2) 6 minutes deposition without/with grain overlay. (c1, c2) 18 minutes deposition without/with grain overlay.

For 15s TMA deposition, only a single orientation was visible in acTEM imaging mode, indicating that the domain sizes can exceed 150×150 nm^2 (the area visible to the CCD at the magnification used). For 1, 6 and 18 minute depositions two grains of different orientation

relative to the underlying graphene were visible. The typical domain size for each film thickness is summarized below:

**Deposition Time**	**Domain Sizes (Height × Width) / nm^2**
15 s	>150×150 (greater than acTEM image frame)
1 minute	(50±10)×(50±10)
6 minutes	(60±10)×(60±10)
18 minutes	(50±10)×(50±10)

Table S1. acTEM grain orientation map sizes for different thickness TMA. Width and height of grains were measured directly from overlaid grain maps.

S7: Mapping the orientation of grains in the TPA thin films

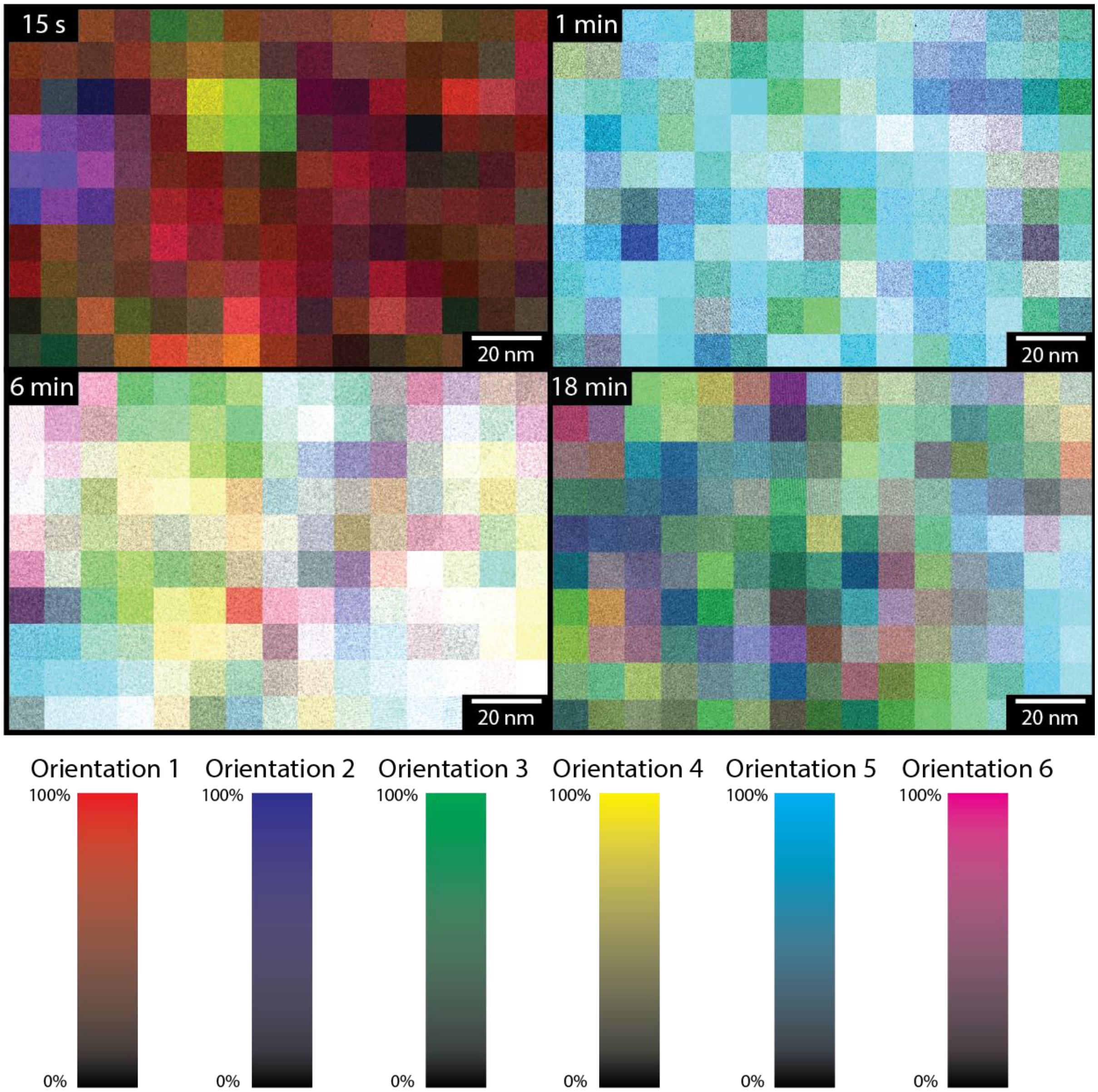

Figure S7. acTEM grain orientation maps for different thickness TPA. Different coloured ‘pixels’ are overlaid on the original acTEM image to indicate regions with one preferential grain orientation. Regions of white present in the 6 minute film indicate approximately equal contributions from all orientations.

S8: *Multislice acTEM image simulations of TMA thin films*

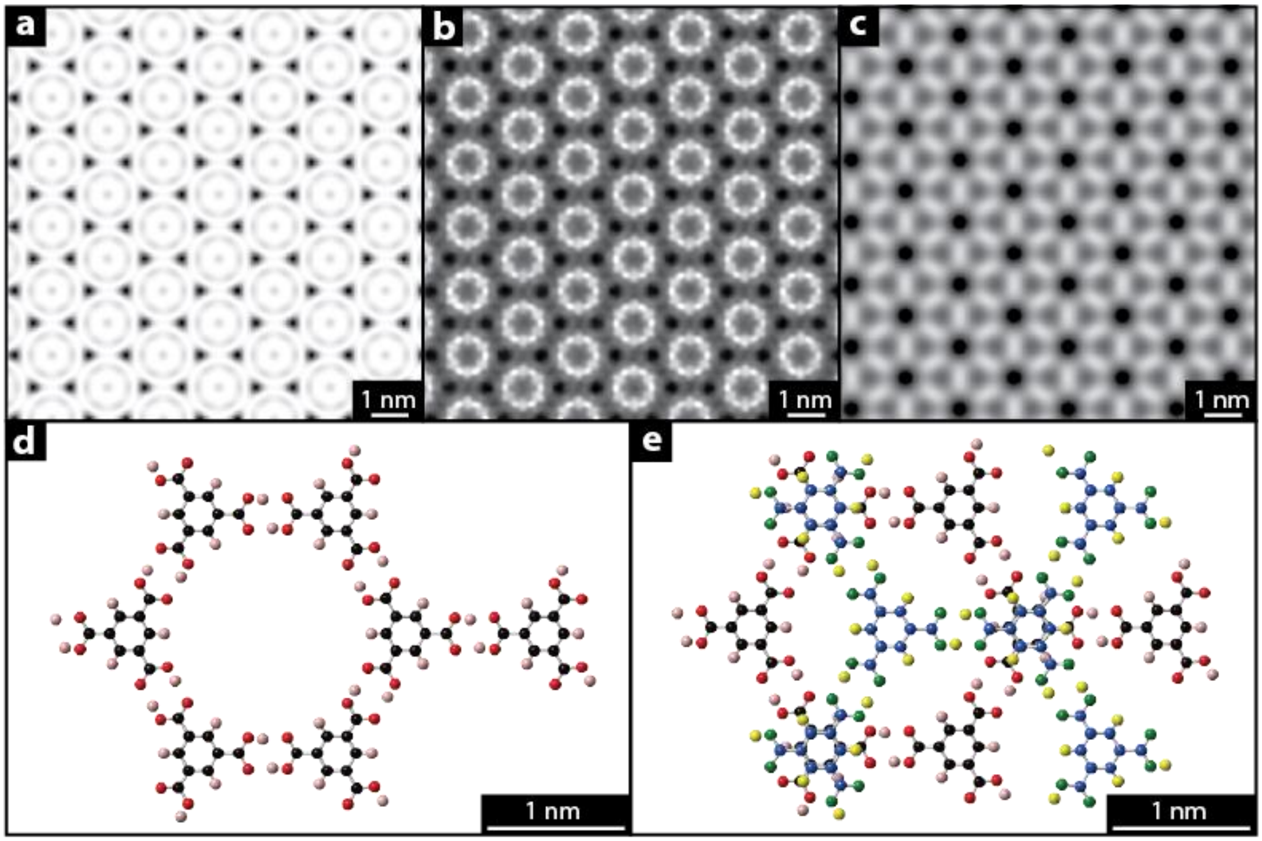

Figure S8a. TMA acTEM reconstructed image (a) and multislice image simulations for direct stacking (b) and offset stacking models (c) for 1 minute deposition thickness. Molecular models for direct stacking (d) and offset stacking (e) used for the multislice simulations. Multislice simulations were performed using spherical aberration $-1\mu m$ and defocus –40nm (as measured for imaging). Simulations were performed using clTEM, an open-source multislice simulation package (M. A. Dyson, clTEM: OpenCL TEM/STEM simulation code, URL ⟨http://github.com/ADyson/clTEM⟩, 2014).

The simulated image assuming direct stacking (AA stacking) is clearly more consistent with the reconstructed one, than the simulated image based on an offset model (AB stacking).

acTEM imaging thus proves that the TMA is stacking in a direct (AA) fashion, creating nanopores.

TEM image reconstruction background

For an electron wavefunction propagating through a unit cell with N atoms located at positions $\underline{\boldsymbol{a}}_j$, where $j = 1, \dots, N$, the electrostatic potential $\varphi(\underline{\boldsymbol{r}})$ felt by the electron at a point $\underline{\boldsymbol{r}}$ may be determined through a summation of all potentials at point $\underline{\boldsymbol{r}}$:

$$\varphi(\underline{\boldsymbol{r}}) = \sum_{j=1}^{N} \varphi_j(\underline{\boldsymbol{r}} - \underline{\boldsymbol{a}}_j).$$

Here, φ_j are the individual potentials of each atom. The potential $\varphi(\underline{\boldsymbol{r}})$ is a continuous real-space function. The Fourier components $\Phi(\underline{\boldsymbol{u}})$ of potential $\varphi(\underline{\boldsymbol{r}})$ are related by:

$$\Phi(\underline{\boldsymbol{u}}) = \mathcal{F}\{\varphi(\underline{\boldsymbol{r}})\} = \frac{1}{V}\int_V \varphi(\underline{\boldsymbol{r}}) e^{-i2\pi(\underline{\boldsymbol{u}}\cdot\underline{\boldsymbol{r}})} \mathrm{d}\underline{\boldsymbol{r}}^3.$$

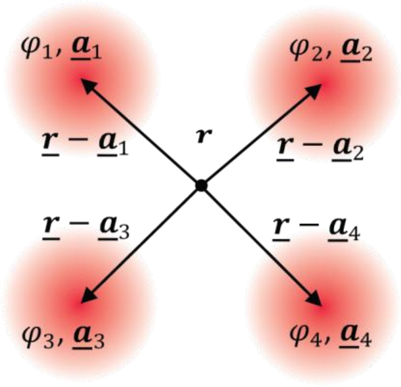

Figure S6b. Reconstruction theory model. Atoms with electrostatic potentials $\varphi_{1,2,3,4}$ are located at coordinates $\underline{\boldsymbol{a}}_{1,2,3,4}$

These $\Phi(\underline{\boldsymbol{u}})$ are related to the structure factor $\mathrm{F}(\underline{\boldsymbol{u}})$ by a scaling factor:

$$\Phi(\underline{\boldsymbol{u}}) = \frac{h^2}{2\pi m_e V}\mathrm{F}(\underline{\boldsymbol{u}}),$$

where the structure factor $F(\underline{\boldsymbol{u}})$ is defined as a discrete sum of structure factor amplitudes $|F_j(\underline{\boldsymbol{u}})|$ and phases $e^{i2\pi(\underline{\boldsymbol{u}}\cdot\underline{\boldsymbol{a}}_j)}$ for each atom in the unit cell:

$$F(\underline{\boldsymbol{u}}) = \sum_{j=1}^{N} |F_j(\underline{\boldsymbol{u}})| e^{i2\pi(\underline{\boldsymbol{u}}\cdot\underline{\boldsymbol{a}}_j)}.$$

Individual structure factor amplitudes and phases are measured in a Fourier transform (power spectrum) of an image of a unit cell or multiple unit cells (these appear as the lattice spots in the power spectrum). The original unit cell potential $\varphi(\underline{\boldsymbol{r}})$ is therefore related to the structure factor $F(\underline{\boldsymbol{u}})$ for the unit cell through a scaled inverse Fourier transform:

$$\varphi(\underline{\boldsymbol{r}}) = \mathcal{F}^{-1}\{F(\underline{\boldsymbol{u}})\} = \frac{h^2}{2\pi m_e V} \sum_{i=1}^{\infty} F(\underline{\boldsymbol{u}}_i) e^{i2\pi(\underline{\boldsymbol{u}}_i\cdot\underline{\boldsymbol{r}})}.$$

Truncating this series to some finite order of $\underline{\boldsymbol{u}}$ gives an approximation to the unit cell potential $\varphi(\underline{\boldsymbol{r}})$:

$$\varphi(\underline{\boldsymbol{r}}) \approx \sum_{i=1}^{M} F(\underline{\boldsymbol{u}}_i) e^{i2\pi(\underline{\boldsymbol{u}}_i\cdot\underline{\boldsymbol{r}})}.$$

This truncation results in a loss of resolution, resulting from the limited resolving power of the microscope and finite number of structure factors present in the image Fourier transform.

Notes on application to real acTEM images: For a acTEM image, image interpretation is difficult due to the effect of lens aberrations on the final image. In addition, multiple scattering occurring due to the large interaction between the incident electrons and sample atoms results in the final image not being a faithful representation of the sample electrostatic potential. Instead, the final image represents the exit-wave of the incident electron beam, convoluted with the various lens aberrations present.[4] These lens aberrations arise due to imperfections in the imaging optics, and may be represented in the form of a contrast transfer function with which the exit wave is convoluted:

$$I = E \otimes CTF,$$

where I is the final image, E is the exit wave and CTF is the contrast transfer function. For particularly thin samples (few atom thick), the exit-wave may be approximated to the projected potential.

***S9:* acTEM imaging of polycrystalline nature of thicker TMA films**

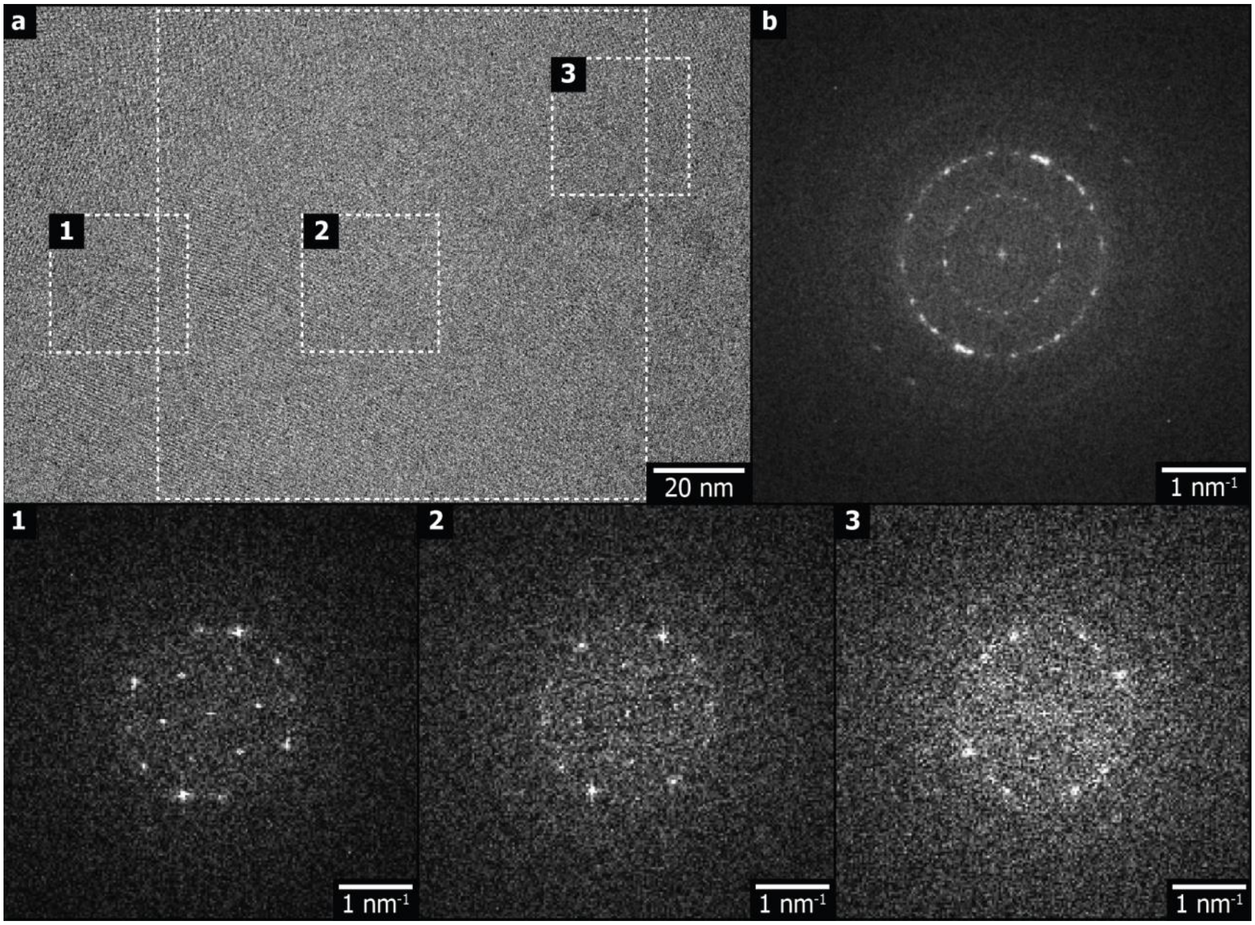

Figure S9. acTEM of the 18 minute-deposition of TMA. The image (a) and the FFT (b) from the large white box demonstrate the polycrystalline nature of the TMA at this thickness. 1,2, and 3 are FFTS from the boxes as labelled in (a).

Figure S9(a) shows an acTEM image of an 18 minute-deposition of TMA on graphene. Periodic features are apparent, as also observed for thinner films, resulting in clear spots in the FFT (b) of the large white box in (a). The FFT shows spots that have the same spacings as seen for lower coverage, suggesting the presence of TMA in a chicken-wire structure. However, there are now several hexagonal arrays present, indicating multiple in-plane orientations, in agreement with diffraction patterns from the same films.

Selected area FFTs can help revealing the structure at smaller lengthscales. In the FFT of region 1, a single set of hexagonal spots is clear, but with another orientation weakly present. This suggests that the templated structure is still dominant in some small regions. However, in most places more than one orientation is visible, as demonstrated by FFTs in regions 2 and 3. The two preferred orientations ($6.8\pm0.1°$ either side of the graphene orientation) are not dominantly visible in the diffraction patterns of the 18 minute-deposition film. As shown in figure S3, this suggests that somewhere between the 6 and 9 minute-deposition point, new and random in-plane orientations appear in small domains, rather than just a continuation of the templating of the original orientations. These new orientations also appear to template upwards, as shown by the presence of discrete spots in the FFTs of the 18 minute-deposition: a non-templated polycrystalline film would present itself as rings or arcs in the acTEM image FFTs.

***S10:** Demonstration of the 6 orientations of TPA apparent by SAED*

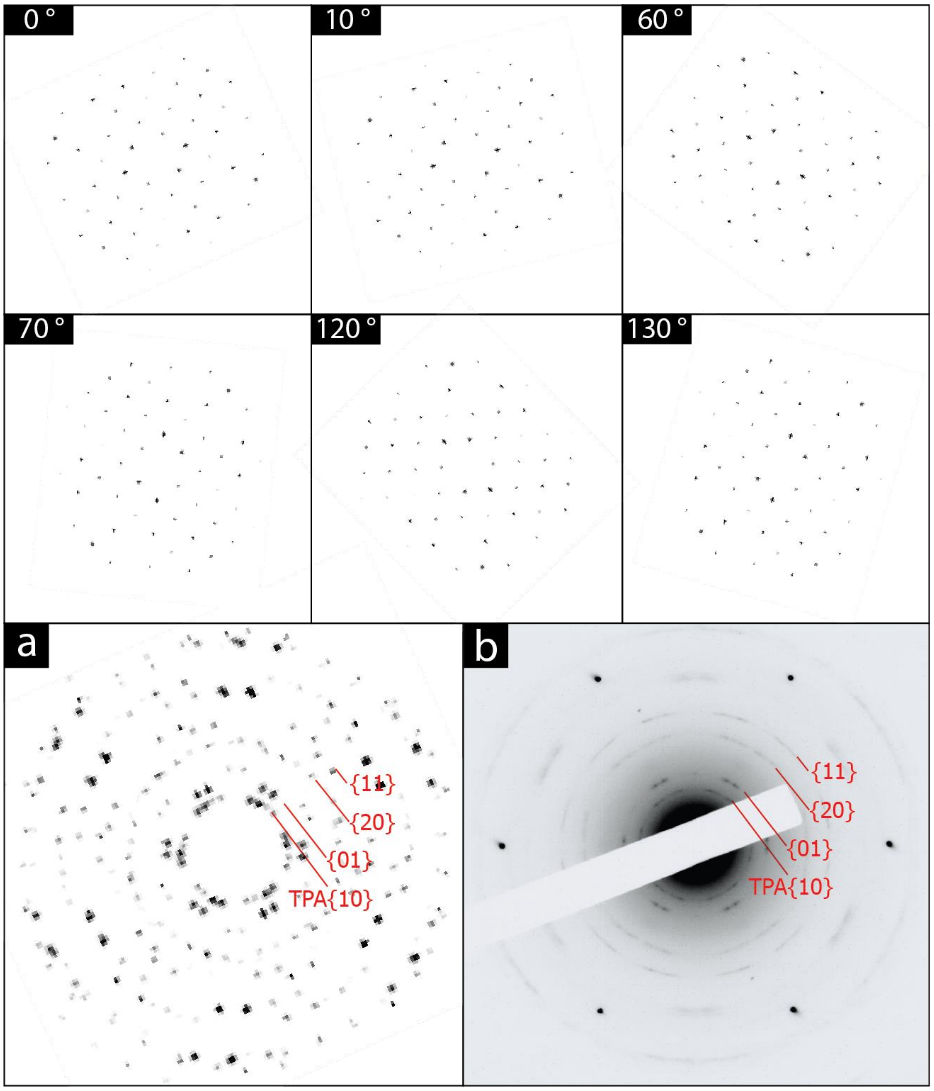

Figure S10. Comparison between simulated diffraction patterns and experimental measurements for 15s TPA deposition. Simulated electron diffraction patterns, assuming the 2D brickwork structure, are shown at six different orientations as labelled. The sum of these simulations is presented in (a) and shows good correspondence with the experimental pattern shown in (b). This indicates the presence of six TPA grains within the selected area used for diffraction. Simulations were performed using clTEM, an open-source multislice simulation package (M. A. Dyson, clTEM: OpenCL TEM/STEM simulation code, URL ⟨http://github.com/ADyson/clTEM⟩, 2014).

S11: *acTEM imaging of TPA thin films*

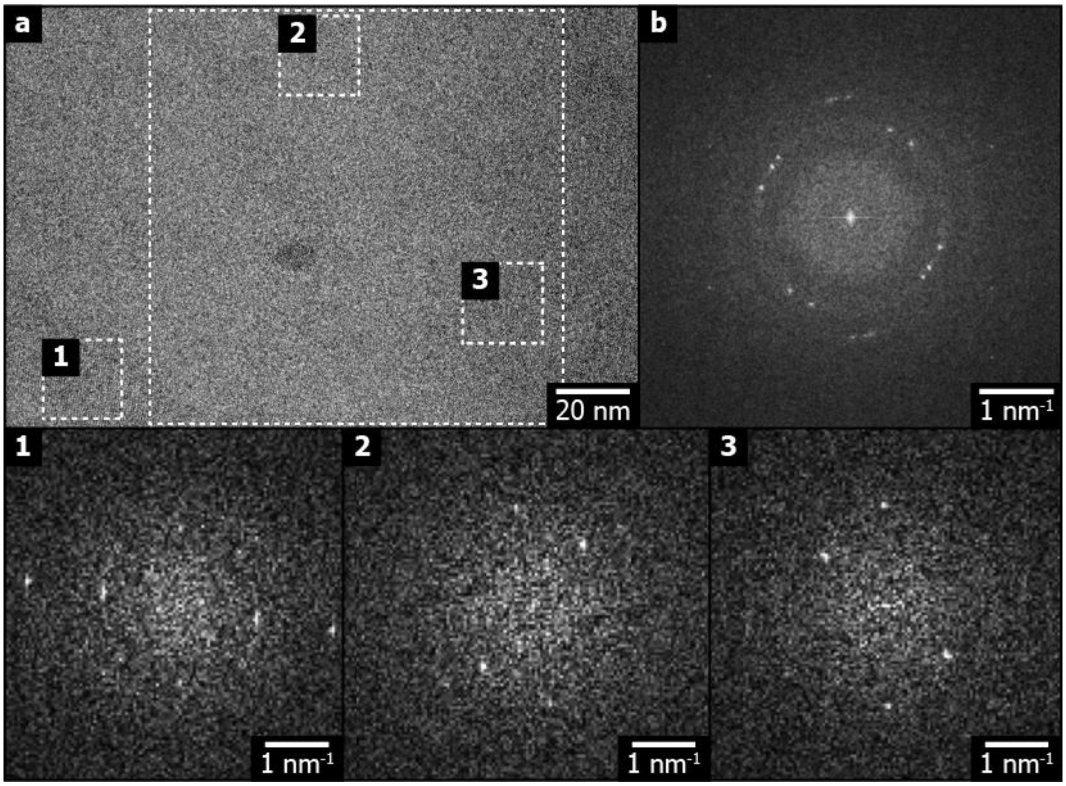

Figure S11a. acTEM of the 1 minute-deposition of TPA. Brightfield image, (a), and corresponding FFTs from the large dashed box, (b), and the smaller dashed boxes 1, 2 and 3 as labelled.

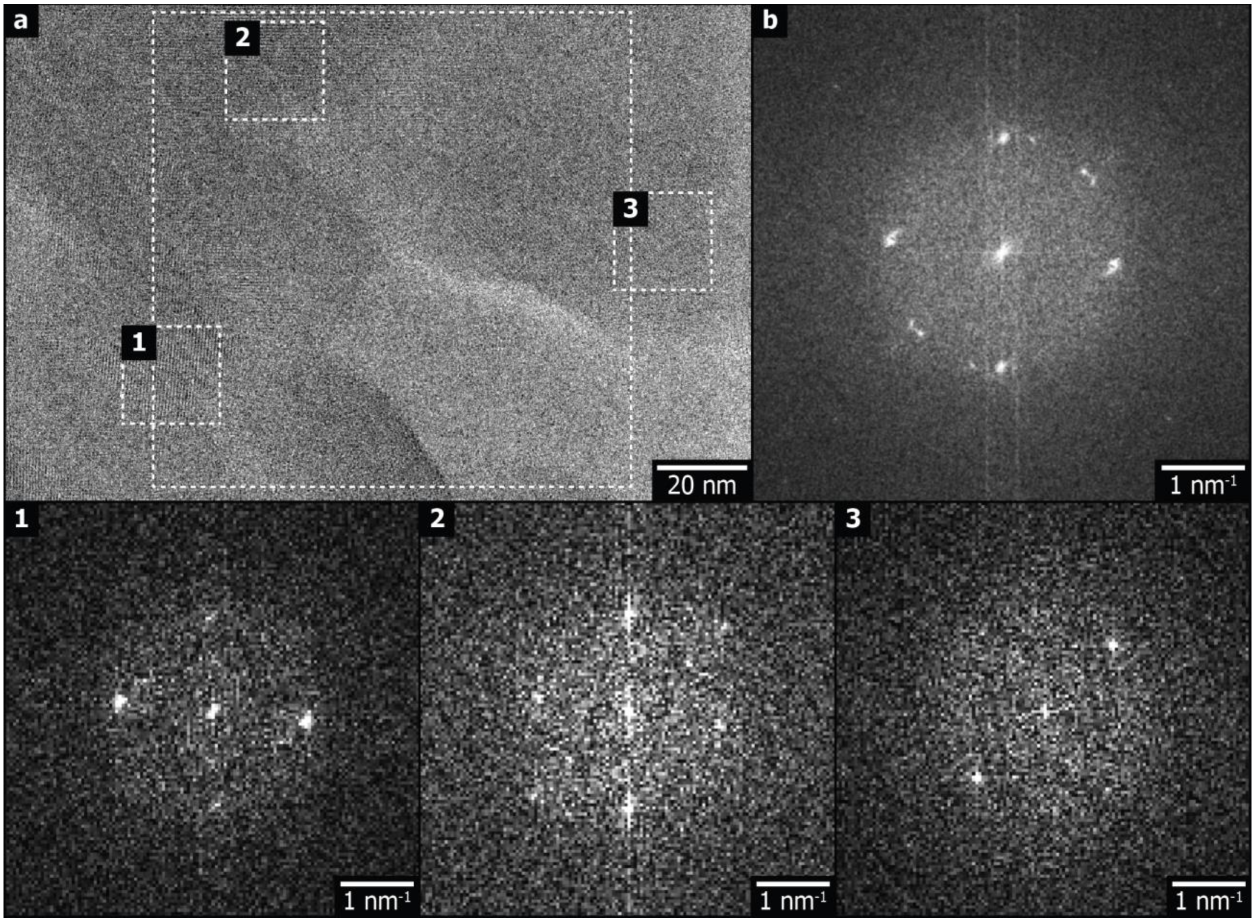

Figure S11b. acTEM of the 18 minute-deposition of TPA. Brightfield image, (a), and corresponding FFTs from the large dashed box, (b), and the smaller dashed boxes 1, 2 and 3 as labelled.

Figures S11a and S11b show acTEM images of 1 minute and 18 minute-depositions of TPA on graphene respectively. The spacings of peaks in the FFT are consistent with the corresponding diffraction patterns shown in Figure 5 of the main article. FFTs of smaller sub-regions show the ordering across the film, demonstrating that the 18 minute deposition is polycrystalline as opposed to forming large single-crystal domains. The angle γ reported in Table 2 is measured from FFTs such as these.

References

1 R. F. Egerton, *Microsc. Res. Tech.*, 2012, **75**, 1550–1556.

2 R. F. Egerton, P. Li and M. Malac, *Micron*, 2004, **35**, 399–409.

3 R. F. Egerton, *Ultramicroscopy*, 2013, **127**, 100–108.

4 X. Zou, S. Hovmoller and P. Oleynikov, *Electron Crystallography: Electron Microscopy and Electron Diffraction*, Oxford University Press, 2011.
